# Transcriptome sequencing in pediatric acute lymphoblastic leukemia identifies fusion genes associated with distinct DNA methylation profiles

**DOI:** 10.1186/s13045-017-0515-y

**Published:** 2017-08-14

**Authors:** Yanara Marincevic-Zuniga, Johan Dahlberg, Sara Nilsson, Amanda Raine, Sara Nystedt, Carl Mårten Lindqvist, Eva C. Berglund, Jonas Abrahamsson, Lucia Cavelier, Erik Forestier, Mats Heyman, Gudmar Lönnerholm, Jessica Nordlund, Ann-Christine Syvänen

**Affiliations:** 1 Department of Medical Sciences, Molecular Medicine and Science for Life Laboratory, Uppsala University, Uppsala, Sweden; 2 Department of Pediatrics, Institution for Clinical Sciences, Sahlgrenska Academy, University of Gothenburg, Gothenburg, Sweden; 3 Clinical Genetics, Uppsala University Hospital, Uppsala, Sweden; 4 Department of Medical Biosciences, University of Umeå, Umeå, Sweden; 5 Karolinska Institutet, Childhood Cancer Research Unit, Astrid Lindgren Children’s Hospital, Karolinska University Hospital, Stockholm, Sweden; 6 Department of Women’s and Children’s Health, Pediatric Oncology, Uppsala University, Uppsala, Sweden

**Keywords:** Pediatric acute lymphoblastic leukemia, RNA sequencing, Fusion genes, BCP-ALL, T-ALL, Translocation

## Abstract

**Background:**

Structural chromosomal rearrangements that lead to expressed fusion genes are a hallmark of acute lymphoblastic leukemia (ALL). In this study, we performed transcriptome sequencing of 134 primary ALL patient samples to comprehensively detect fusion transcripts.

**Methods:**

We combined fusion gene detection with genome-wide DNA methylation analysis, gene expression profiling, and targeted sequencing to determine molecular signatures of emerging ALL subtypes.

**Results:**

We identified 64 unique fusion events distributed among 80 individual patients, of which over 50% have not previously been reported in ALL. Although the majority of the fusion genes were found only in a single patient, we identified several recurrent fusion gene families defined by promiscuous fusion gene partners, such as *ETV6*, *RUNX1*, *PAX5*, and *ZNF384*, or recurrent fusion genes, such as *DUX4-IGH*. Our data show that patients harboring these fusion genes displayed characteristic genome-wide DNA methylation and gene expression signatures in addition to distinct patterns in single nucleotide variants and recurrent copy number alterations.

**Conclusion:**

Our study delineates the fusion gene landscape in pediatric ALL, including both known and novel fusion genes, and highlights fusion gene families with shared molecular etiologies, which may provide additional information for prognosis and therapeutic options in the future.

**Electronic supplementary material:**

The online version of this article (doi:10.1186/s13045-017-0515-y) contains supplementary material, which is available to authorized users.

## Background

Chromosomal rearrangements giving rise to fusion genes play a central role in the malignant transformation of many cancers, including acute lymphoblastic leukemia (ALL) [1]. Recurrent large-scale structural rearrangements that result in expressed fusion transcripts are a hallmark of ALL and are included in the predictors of clinical outcome of individual patients that form the basis for treatment stratification [2]. The well-known subgroups of pediatric ALL based on expressed fusion genes include *BCR-ABL1*, *ETV6-RUNX1*, *TCF3-PBX1*, and 11q23/*MLL* rearrangements, such as *KMT2A-AFF1*, *KMT2A-MLLT3*, *KMT2A-MLLT1*, and *KMT2A-MLLT10*. In the Nordic countries, these fusion genes are routinely screened for at ALL diagnosis using fluorescent in situ hybridization (FISH) or polymerase chain reaction (PCR)-based assays [3].

In addition to recurrent rearrangements, ALL cells typically harbor other chromosomal aberrations that are detectable by routine cytogenetic screening and are non-recurrent or have not yet been associated with expressed fusion genes. Moreover, additional copy-neutral or cryptic translocations or inversions, which are not detectable by clinical routine methods, may occur in ALL cells. Therefore, there are potentially many fusion genes that have so far remained undetected in ALL. Identifying new fusion genes is important as they can serve as novel therapeutic targets and provide prognostic information [4]. Recent developments in transcriptome sequencing have enabled precise and sensitive detection of fusion genes in T cell acute lymphoblastic leukemia (T-ALL) [5, 6] and in B cell precursor acute lymphoblastic leukemia (BCP-ALL) [4, 7–13]. The most significant discoveries are the characterization of in-frame fusion genes involving *DUX4*, *ZNF384*, and *MEF2D*, which each define a new molecular subgroup of pediatric ALL with a distinct gene expression profile [12, 14, 15]. However, there is large heterogeneity regarding the cytogenetic subtypes screened and type of fusion genes that have been reported in these studies.

In the current study, we performed systematic analysis of expressed fusion genes by transcriptome sequencing in BCP-ALL and T-ALL cells collected at diagnosis from 134 patients with pediatric ALL. We surveyed the fusion gene landscape in 74 BCP-ALL patients with well-characterized recurrent subtypes, 42 BCP-ALL patients with karyotypes denoted “other” or “normal” that lack a defined cytogenetic subtype at diagnosis (BCP-ALL “other”), and 18 T-ALL patients. In total, we identified and validated 64 unique fusion events in 80 of the patients of which several have not been previously observed in pediatric ALL. We also identified distinct DNA methylation and gene expression profiles associated with recurrent fusion genes.

## Methods

### Patient and control samples

Bone marrow aspirates or peripheral blood samples were collected at diagnosis from 134 pediatric ALL patients. ALL diagnosis was established by analysis of leukemic cells with respect to morphology, immunophenotype, and cytogenetics (Table 1). The samples included in the study were of B cell precursor (BCP-ALL; *n* = 116) or of T cell immunophenotype (T-ALL; *n* = 18). Lymphocytes were isolated from the samples by Ficoll-isopaque centrifugation, and the proportion of leukemic blasts was determined by light microscopy as previously described [16]. The samples selected for analysis contained at least 80% leukemic blasts (average 91%). High-quality RNA was extracted with the AllPrep DNA/RNA Mini Kit (QIAGEN). No systematic differences in blast count or RNA quality was observed between the bone marrow aspirates and peripheral blood samples. The majority of patients were treated according to Nordic Society of Pediatric Hematology and Oncology (NOPHO) protocols except for patients with t(9;22)*BCR-ABL1* who were treated with the EsPh-ALL protocol and children below 1 year who were treated according to Interfant protocols [3, 17, 18] (Additional file 1: Table S1). RNA was extracted from normal CD19^+^ B cells (*n* = 5) and CD3^+^ T cells (*n* = 5) isolated from peripheral blood mononuclear cells from five healthy Swedish blood donors as described previously [19]. Additional details about the RNA samples, characteristics of the leukemic cells, and treatment protocols can be found in Additional file 2. The guardians of the patients provided written or oral consent to the study. The study was approved by the regional ethics board in Uppsala, Sweden.

**Table 1:** Summary of ALL samples included in the study

Immuno-phenotype	Cytogenetic abnormality	Fusion gene	*N*	Median WBC at diagnosis, × 10^9/L (range)	Median age at diagnosis, years (range)
T-ALL	Various		18	173.5 (1–588)	11.9 (1.9–16.8)
BCP-ALL	HeH	–	42	9.6 (0.8–124)	3.6 (1.0–17.74
	t(12;21)	*ETV6*-*RUNX1*	18	6.5 (1.1–95)	4.9 (2.2–12.7)
	11q23/*MLL*	*MLL*-r	7	193 (1.8–744)	0.6 (0.5–1.7)
	t(9;22)	*BCR*-*ABL1*	6	88.3 (14.1–180)	12.1 (9.3–13.5)
	dic(9;20)	–	1	41.8 (41.8–41.8)	4.4 (4.4–4.4)
	Various (BCP-ALL “other”)^a^	–	42	7.9 (0–213)	8.8 (1.4–17.7)
*Total*			*134*		

^a^BCP-ALL samples negative for targeted assays for known ALL cytogenetic aberrations with either non-recurrent aberrations, normal karyotypes, or no results available from cytogenetic analysis

*WBC* white blood cell count at diagnosis, *MLL*-r rearrangements involving the KMT2A (MLL) gene

### Library preparation and transcriptome sequencing

Strand-specific RNA sequencing libraries were constructed from rRNA-depleted RNA using the ScriptSeq V2 Kit according to the manufacturer’s instructions (Epicentre). The libraries were sequenced using a Hiseq2000/2500 instrument (Illumina), 50 bp paired-end, with the exception of one sample (ALL_707) that was sequenced on a MiSeq instrument, 83 bp paired-end. Raw sequence reads were trimmed using Cutadapt 1.2.1 [20] and mapped to the human 1000 Genomes build 37 (GRCh37) using Tophat 2 (2.0.4) [21]. Quality control of RNA sequencing data was performed with RNA-SeQC [22] (Additional file 1: Table S2). Detailed information of RNA sequencing and computational analysis is provided in Additional file 2.

### Fusion gene detection

Fusion genes were detected using a two-pronged approach. First, FusionCatcher v0.99.4a beta was used to screen for novel and known fusion genes in an unbiased manner (de novo search) [23]. Next, we screened a set of predefined established fusion genes in pediatric ALL (targeted search) by counting uniquely aligned reads supporting the fusion gene (Additional file 3: Fig. S1, Additional file 1: Table S4). To reduce false positives, a filtering process of the fusion genes detected by FusionCatcher was applied. These measures consisted of removing fusion genes that met any of the following criteria: blacklisted fusion genes based on reference data provided by FusionCatcher (Additional file 1: Table S3), fusion genes with fusion-supporting reads that map to multiple genomic locations indicative of sequence homology (common mapping reads), fusion genes where the 5′ or the 3′ fusion gene partner mapped to many other genes (highly promiscuous gene) (Additional file 1: Table S3), and fusion genes with less than three unique sequencing reads that support a detected chimeric transcript. Genes previously described to be involved in ALL-related fusion events were retained throughout all filtering steps. All fusion genes were experimentally validated by Sanger sequencing (Additional file 1: Table S5, Additional file 3: Fig. S1). Additional information about the fusion gene detection, the filtering procedure, and the validation is provided in Additional file 2.

### Gene expression profiling

For quantification of gene expression, counts of aligned reads were summarized using featureCounts [24] and normalized using variance stabilizing transformation (*R* package DESeq2) [25]. Gene expression levels in fragments per kilobase per million mapped reads (FPKM) were determined using Cufflinks version 2.2.0 [26], and Cuffdiff was subsequently used to detect differentially expressed genes [27]. Analysis of differential gene expression was performed by pair-wise comparison of the expression levels between each of the following subgroups: *DUX4*-*IGH* (*n* = 8), *ZNF384* rearrangements (*n* = 6), t(12;21)*ETV6*-*RUNX1* (*n* = 18), t(9;22)*BCR*-*ABL1* (*n* = 6), 11q23/*MLL* (*n* = 7), high hyperdiploid (HeH) (*n* = 42), and normal CD19^+^ B cells (*n* = 5). For determining differentially expressed genes in the groups with *DUX4*-*IGH* and *ZNF384* rearrangements, only pair-wise comparisons with a false discovery rate corrected *p* value less than 0.1 and twofold difference in mean expression detected between three or more compared groups were regarded as differentially expressed. Lowly expressed genes with average FPKM < 5 were excluded from the analysis.

### DNA methylation analysis

Previously published genome-wide DNA methylation data for ~450,000 CpG sites (450 k) was available for 130 out of the 134 patients included in the present study (GSE49031) [19]. Differentially methylated CpG sites (DMCs) were determined as previously described [19]. Briefly, DMCs were determined in the *DUX4*-*IGH*- and *ZNF384*-rearranged subgroups using remission bone marrow, CD19^+^ B cells, and CD34^+^ progenitor cells as the reference. A minimal cut-off value of 0.2 was applied for the mean absolute difference in DNA methylation (∆β) to highlight CpG sites with large differences between groups. The *R* package “CopyNumber450kCancer” was used to detect copy number alterations with the 450 k data [28].

### Detection of somatic variants

Data from previously performed mutational analysis was available for 75 of the patients included in the present study [29]. Briefly, the exons of 872 cancer genes were captured using a HaloPlex Target Enrichment panel and sequenced to high depth (> 500×). DNA from matched remission samples and from healthy Swedish blood donors were used as controls. Single nucleotide variants (SNVs) were called using Freebayes and putative SNVs were filtered and annotated as previously described [29, 30].

### Rapid amplification of cDNA ends

Rapid amplification of cDNA ends (RACE) was performed using the SMARTer RACE 5′/3′ Kit (Clontech) with 250 ng total RNA as input. The primers used for 3′ RACE of *DUX4* and 5′ RACE of *ZNF384* can be found in Additional file 1: Table S5. Sequencing libraries were prepared from the RACE fragments using the MicroPlex Library Preparation Kit v2 kit (Diagenode). Libraries were pooled and sequenced on a MiSeq instrument, PE150 bp read length using V2 chemistry. The reads were aligned to GRCh37 using Tophat 2 (2.0.4) [21] and STAR [31], and data were visualized in IGV [32].

### Data availability

RNA sequencing data are available for academic purposes by contacting the corresponding author, as the patient/parent consent does not cover depositing data that can be used for large-scale determination of germline variants.

## Results

### Transcriptome sequencing and detection of fusion genes

**Fig. 1:**
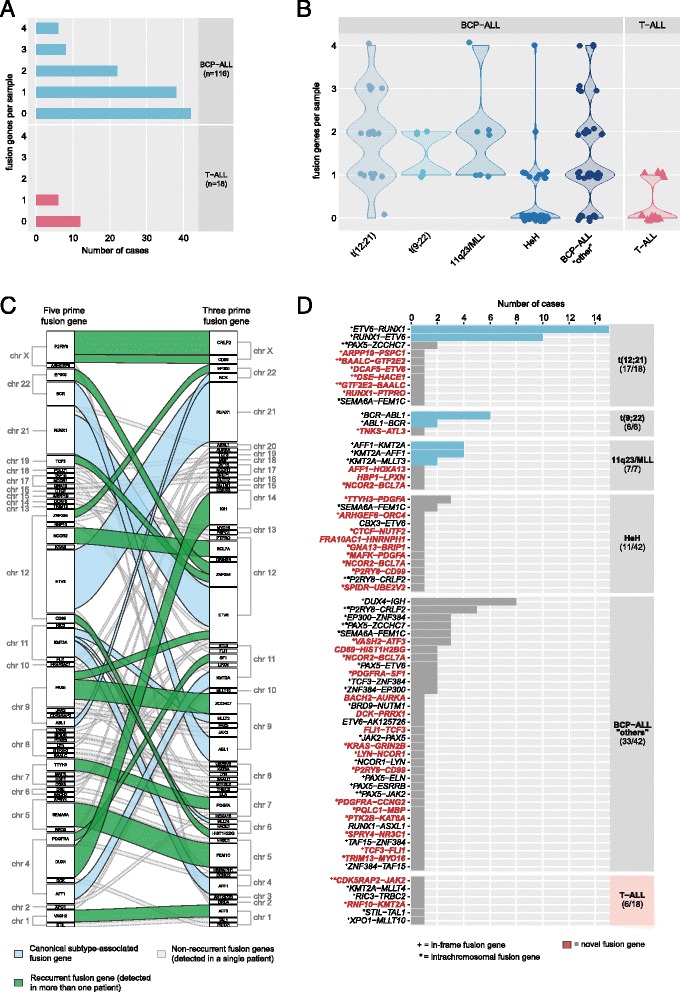
Distribution of validated fusion transcripts among 134 ALL patients. **a** Number of fusion genes detected per sample in BCP-ALL and T-ALL patients. The vertical axis shows the number of fusion genes identified per sample ranging from zero (no detectable fusion gene) up to four co-expressed fusion genes. **b** Violin plots showing the number of fusion genes per patient in each ALL subtype. No fusion genes were identified in the single dic(9;20) patient, and thus, this sample is excluded from the plot. The vertical axis shows the number of fusion genes identified per sample. Each dot represents one patient. **c** Chromosomal location of the 64 unique fusion events. The 5′ fusion gene partners are plotted in the left panel, and the 3′ fusion gene partners are plotted in the right panel together with their respective chromosomal locations. The thickness of the connecting lines reflects the recurrence of the fusion gene. *Blue lines* represent the canonical fusion genes associated with the t(12;21), t(9;22), or 11q23/*MLL* subtypes. *Green lines* represent recurrent non-canonical fusion genes and dashed gray lines represent fusion genes identified in a single patient. **d** Number of fusion genes per ALL subtype. The *bars* indicate the number of patients in which a given fusion gene was observed by subtype. The panel to the right of the plot shows the number of patients with any fusion gene out of all of patients belonging to the given subtype. The canonical fusion genes are highlighted in *blue*. Novel fusion genes discovered in this study are highlighted in red. + = in-frame fusion events. * = intra-chromosomal fusion events

To identify fusion genes in pediatric ALL, we sequenced the transcriptomes of 134 ALL samples, including 116 BCP-ALL and 18 T-ALL patients (Table 1; Additional file 1: Table S1). RNA sequencing yielded between 19 and 120 million (average 46 million) paired-end reads per sample. We detected 2136 candidate fusion transcripts with the FusionCatcher software across all the samples prior to filtering [23]. On average, we discovered 31 candidate fusion genes per sample (range 2–158). To reduce the number of potential false-positive fusion transcripts, we performed stringent filtering of the candidate fusion transcripts, including filtering of fusion genes called in the normal B and T cell to enrich for cancer-specific fusion genes in the ALL samples. This filtering procedure rendered a set of 197 unique candidate fusion genes identified in 97 of the ALL patient samples (Additional file 3: Fig. S1B). Next, we validated candidate fusion genes by visual examination of the aligned sequencing reads that supported a fusion junction in the RNA sequencing data. Of these candidates, 104 were selected for further validation by PCR followed by Sanger sequencing, where 61 of the fusion genes were experimentally validated (Additional file 1: Table S6). In addition, we performed targeted screening of 22 well-established ALL fusion genes whereby additional fusion genes were detected, including *DUX4-IGH* (*n* = 8 patients), *TAF15-ZNF384* (*n* = 1), and *STIL-TAL1* (*n* = 1) (Additional file 1: Table S4). Thus, after filtering and experimental validation, we detected a total of 64 unique fusion events, corresponding to 136 fusion genes in 80 of the patients included in the study (Fig. 1).

### Characteristics of the fusion genes in pediatric ALL

A fusion gene was detected in 74 of the 116 BCP-ALL patients, whereas a fusion gene was detected in only six out of the 18 T-ALL patients analyzed. No difference in fusion gene calls was observed between samples originating from the bone marrow or peripheral blood. Among the BCP-ALL patients with a detectable fusion gene, the frequency of fusion genes varied from one to four co-occurring fusion genes, although in the majority of the BCP-ALL patients (38/74), only one fusion gene was detected per sample (Fig. 1a). In the six T-ALL patients with a detectable fusion gene, only a single fusion gene was observed per sample.

In the BCP-ALL subtypes t(12;21)*ETV6-RUNX1*, t(9;22)*BCR-ABL1*, and 11q23/*MLL*, a fusion gene was detected by RNA-sequencing in all but one t(12;21) patient (ALL_504) and patients with the HeH subtype had the lowest frequency of fusion genes (Fig. 1b). In addition to fusion genes that are characteristic for ALL, we identified several other fusion genes in our patient cohort (Fig. 1c, d). The largest proportion of unique fusion genes was detected in the BCP-ALL “other” subgroup where a fusion gene was detected in 33 of the 42 patients, of which 16 patients expressed more than one.

Forty of the 64 unique fusion genes occurred between two genes on different chromosomes, while 24 fusion genes were caused by presumptive intra-chromosomal rearrangements, of which 18 (75%) of the involved genes are located over 1 Mbp distance from each other (Fig. 1c, d, Additional file 1: Table S6). Thirty-six fusion genes had an open reading frame, and in-frame fusion genes were more common in t(12;21), t(9;22), or the 11q23/*MLL* than in the ALL patients with HeH, where only two out of 12 fusion genes were in-frame. Most fusion genes (43/64) were only detected in a single patient and 36 (56%) have not been previously described in ALL**.**

The most common fusion gene was the well-known *ETV6-RUNX1* and its reciprocal *RUNX1-ETV6* in the t(12;21) BCP-ALL subtype (Fig. 1d). We also detected eight fusion genes, including six novel in-frame fusion genes that were expressed concurrently with *ETV6*-*RUNX1*. Contrary to the t(12;21) subtype, we only identified a single in-frame fusion gene (*TNKS-ATL3*) in addition to the canonical *BCR-ABL1*/*ABL1-BCR* in the t(9;22) subtype. In the 11q23/*MLL* subtype, no in-frame fusion genes were detected besides the canonical *KMT2A*-*AFF1*/*AFF1*-*KMT2A* and *KMT2A*-*MLLT3*. Among the BCP-ALL “other” patients, *DUX4-IGH* was detected in eight out of 42 patients and was thus the most common fusion gene in this subgroup, followed by recurrent fusion events involving *ZNF384* or *PAX5*.

### Genomic breakpoints

Information from karyotyping performed at diagnosis provided support for genomic breakpoints giving rise to the fusion genes. In addition to the canonical fusion genes in t(12;21), t(9;22), and 11q23/*MLL*, we found evidence for genomic breakpoints for 12 fusion genes in the karyotype data. Supporting evidence in the karyotype data was primarily observed as translocations between the chromosomes where the fusion genes are located (Additional file 1: Table S7). Furthermore, we used array-based copy number analysis (CNA) to detect evidence for chromosomal rearrangements within or in close proximity of the genes involved in a fusion event. Deletions or amplifications in the genomic regions of genes involved in a fusion event were identified for an additional 11 fusion genes (Additional file 1: Table S7, Additional file 3: Fig. S2). Thus, in total, we detected the presumable genomic breakpoint that gave rise to 23 out of the 57 unique non-canonical fusion genes.

### Recurrent fusion genes

**Fig. 2:**
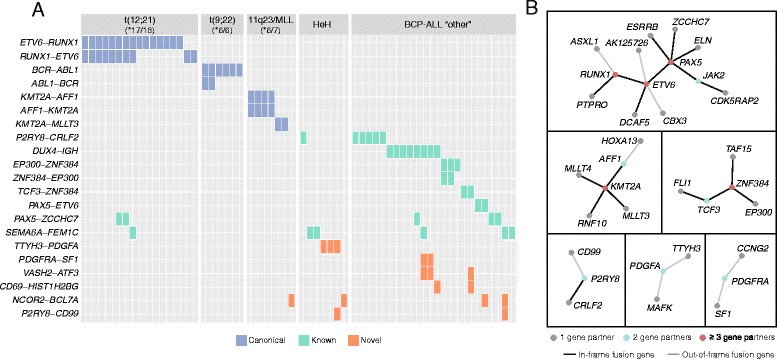
Recurrent fusion gene families in ALL. **a** Twenty-one fusion genes were recurrent in BCP-ALL patients. The fusion genes are plotted along the vertical axis, and the patients are plotted along the horizontal axis by the subtype given at ALL diagnosis. The *numbers* in the *upper panels* indicate the number of patients where the expected canonical fusion gene was detected by RNA sequencing in each subtype. The canonical subtype-associated fusion genes are marked in *blue*, the previously reported fusion genes in ALL are indicated in *green*, and the novel fusion genes discovered here are indicated in *red*. **b** Frequently translocated genes giving rise to expressed fusion genes formed six independent node groups. The nodes representing genes that fused with two different partners are shown with *green dots*, and genes that can have three or more fusion partners are shown with *red dots*. In-frame fusion genes are highlighted with a *black line*

Of the 64 fusion genes, 21 were recurrent in BCP-ALL (Fig. 2a). Patients with t(12;21)*ETV6-RUNX1* (*n* = 18), t(9;22)*BCR-ABL* (*n* = 6), and 11q23/*MLL* (*n* = 7), all harbored translocations resulting in fusion genes that had been verified either by FISH or RT-PCR and served as positive controls for fusion gene detection by RNA sequencing. We detected the characteristic subtype-defining fusion transcripts, including their reciprocal fusion genes, in 29/31 (94%) of the patients in this positive control group (Additional file 1: Table S1). The expected canonical fusion gene was not detected in two patients. In both cases (ALL_504 t(12;21) and ALL_16 11q23/*MLL*), the libraries were among those with the lowest sequence depth in the study (32 and 35 million read-pairs, respectively) (Additional file 1: Table S1). The targeted approach to identify fusion-supporting reads for *ETV6-RUNX1* in ALL_504 revealed only two reads, while no fusion supporting reads in ALL_16 were detected for 11q23/*MLL*-related fusion partners, although this does not exclude the presence of an unknown or rare fusion partner that was not included in our target approach. It is highly likely that low sequencing depth in these two cases contributed to the false negative result.

Balanced translocations, which can result in expression of reciprocal fusion genes, occur in the t(12;21)*ETV6-RUNX1*, t(9;22)*BCR-ABL1*, and 11q23/*MLL* subtypes. In agreement with this, we found co-expression of the reciprocal fusion gene in several patients belonging to these three BCP-ALL subtypes (Fig. 2a). In the 11q23/*MLL* subgroup, we found co-expression of *KMT2A-AFF1* and *AFF1-KMT2A* in all four patients with t(4;11), but no evidence for co-expression of the reciprocal fusion gene in the two patients with *KMT2A-MLLT3*. We also identified reciprocal *BCR-ABL1* and *ABL1-BCR* in two out of the six t(9;22) patients. In the t(12;21) subtype, we detected co-expressed *ETV6-RUNX*1 and reciprocal *RUNX1-ETV6* in eight out of 18 t(12;21) patients. Moreover, in three out of the seven patients with *ETV6-RUNX1*, but without the reciprocal *RUNX1*-*ETV6*, we identified co-expression of two other previously unreported in-frame fusion genes, namely *DCAF5-ETV6* (ALL_386) and *RUNX1-PTPRO* (ALL_9 and ALL_678), which appear to have arisen from cryptic unbalanced translocation of the truncated *ETV6* or *RUNX1* gene to another chromosomal region (Additional file 1: Table S1). Interestingly, the *PTPRO* gene is located on chromosome 12 approximately 3.6 Mbp downstream of *ETV6*. The *DCAF5* gene is located on chromosome 14, and the karyotype of ALL_386 revealed a complex translocation involving chromosomes 3, 12, and 14.

The *DUX4-IGH* fusion gene (*n* = 8) and *ZNF384* rearrangements involving *EP300* (*n* = 3) or *TCF3* (*n* = 2) were the most frequently occurring fusion genes in the BCP-ALL “other” group in addition to *P2RY8-CRLF2*. Together with *PAX5-ETV6* (*n* = 2), *DUX4-IGH* and *ZNF384* rearrangements were observed exclusively in BCP-ALL “other” patients. The novel *PDGFRA*-*SF1* (*n* = 2), *VASH2*-*ATF3* (*n* = 3), and *CD69*-*HIST1H2BG* (*n* = 2) were also found exclusively in BCP-ALL “other” and co-expressed with aforementioned fusion genes such as *DUX4*-*IGH* or *ZNF384*-*TCF3*. Interestingly, *TTHY3*-*PDGFA* was found exclusively in three patients with HeH.

### Fusion gene nodes

In addition to the recurrent fusion genes described above, we identified promiscuous genes that were fused with several gene partners (Fig. 2b). Of the 89 unique genes involved in fusion events, 11 genes had more than one fusion partner and constitute six independent nodes. These genes include the well-known ALL genes *ETV6*, *RUNX1*, *KMT2A*, and *PAX5* that form fusion genes with up to five different partners; the emerging BCP-ALL subgroup defined by *ZNF384* rearrangements; and the new *P2RY8*, *PDGFA*, and *PDGFRA* nodes.

*ETV6* and *PAX5* were the most frequently translocated genes and formed the largest network of gene connections often resulting in in-frame fusion genes. Although fusion genes involving *ETV6* and *RUNX1* were predominantly detected in the t(12;21) subgroup, *CBX3*-*ETV6*, *RUNX1*-*ASXL1*, and *ETV6*-*AK125726* were detected in patients with other subtypes. These three out-of-frame fusion genes have been previously described in these patients based on a t(12;21)-like DNA methylation signature [8]. This is contrary to the general pattern in the t(12;21) subgroup, where additional in-frame fusion genes with *ETV6* or *RUNX1* are expected. For example, *DCAF5*-*ETV6* and *RUNX1*-*PTPRO* are both in-frame.

### DNA methylation and transcriptional signatures

**Fig. 3:**
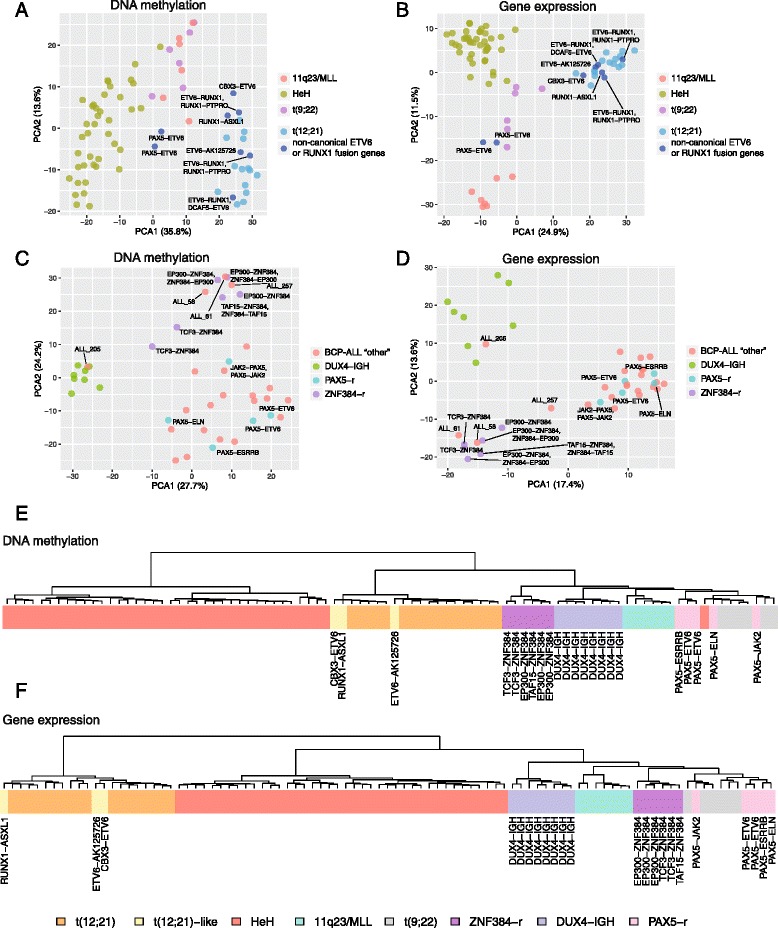
Clustering analysis using genome-wide DNA methylation and gene expression patterns in BCP-ALL patients with expressed fusion genes. **a**, **b** Principal component analysis of patients harboring non-canonical fusion genes involving *ETV6* or *RUNX1* together with patient samples with an established cytogenetic subtype (t(12;21)*ETV6-RUNX1*, t(9;22)*BCR*-*ABL1*, 11q23/*MLL*, and HeH) using **a** DNA methylation and **b** gene expression data. The fraction of the variance explained by principal component 1 (PC1) and principal component 2 (PC2) are shown on the horizontal and vertical axes, respectively. **c**, **d** Principal component analysis of BCP-ALL “other” patients using **c** DNA methylation and **d** gene expression data. Patients with *DUX4*-*IGH* rearrangements, *ZNF384* rearrangements (*ZNF384*-r), and *PAX5* rearrangements (*PAX5*-r), together with BCP-ALL “other” patients harboring other fusion genes or no detectable fusion gene, are shown in the figures. Patients that cluster together with *IGH*-*DUX4*- or *ZNF384*-rearranged patients, although with no characterizing fusion gene, are highlighted in the figure. The fraction of the variance explained by principal component 1 (PC1) and principal component 2 (PC2) are shown on the horizontal and vertical axes, respectively. **e**, **f** Unsupervised hierarchical clustering using **e** DNA methylation and **f** gene expression data. Patients belonging to the t(12;21)*ETV6-RUNX1*, t(9;22)*BCR*-*ABL1*, 11q23/*MLL*, and HeH subtypes are shown together with patients harboring t(12;21)-like fusion genes, *DUX4*-*IGH*, *ZNF384*, and *PAX5* rearrangements. For ease of interpretation, only one of the paired reciprocal fusion genes detected in a sample is shown in panels **e** and **f**. The 1000 most variably methylated CpG sites or expressed genes were used in all panels

Next, to obtain a view of the molecular variation associated with recurrent fusion genes in our dataset, we performed unsupervised clustering analysis using array-based genome-wide DNA methylation and gene expression data from RNA sequencing (Fig. 3, Additional file 3: Fig. S3). T(12;21)-like DNA methylation patterns have previously been described for three of the patients included in this study (ALL_11, ALL_106, and ALL_495) as mentioned above. These patients were found to harbor fusion genes involving either *ETV6* or *RUNX1*, but not the canonical *ETV6*-*RUNX1* fusion gene [8]. In agreement with these findings, these three patients clustered together with the t(12;21)*ETV6*-*RUNX1* patients in DNA methylation and gene expression data (Fig. 3a, b, e, f). Notably, the patient with a *CBX3-ETV6* fusion gene that was diagnosed as HeH and verified by CNA clustered together with the t(12;21) rather than with the HeH subgroup. Furthermore, three t(12;21) patients (ALL_386, ALL_9, and ALL_678) harboring unbalanced t(12;21)-translocations with *DCAF5*-*ETV6* or *RUNX1*-*PTPRO*, and six patients in which only one of the reciprocal fusion genes was detected, clustered with the t(12;21) based on both DNA methylation and gene expression data. The two patients with *PAX5*-*ETV6* did not cluster with the t(12;21) patients, most likely due to the downstream effect of altered *PAX5*, rather than *ETV6* in these patients.

We also examined the DNA methylation and gene expression patterns for recurrent fusion gene families characterized by *DUX4-IGH*, *ZNF384*, or *PAX5* rearrangements in the BCP-ALL “other” group. Three major clusters defined by the recurrent fusion gene families emerged (Fig. 3c, d). One additional patient (ALL_205) clustered together with the eight *DUX4*-*IGH* patients, although *DUX4-IGH* was not detected by RNA sequencing. *DUX4*-rearranged cases have previously been associated with a distinct over-expression of the *DUX4* gene [14]. In agreement with this, ALL_205 displayed similarly high levels of *DUX4* expression as the *DUX4*-*IGH*-positive cases at much higher levels compared to other BCP-ALL subgroups and controls (Additional file 3: Fig. S4A). 3′ RACE confirmed the presence of a *DUX4-IGH* fusion gene in ALL_205; however, ~470 bp from chromosome 8q24.21 (chr8:130,691,944-130,692,413) was identified as inserted between the *DUX4* and *IGH* genes in the fusion transcript; thus, it was not initially detected by our targeted screening approach (Additional file 3: Fig. S5A).

The six patients with *ZNF384* rearrangements clustered together in both the DNA methylation and gene expression data (Fig. 3c–f). In the *ZNF384* cluster, we observed three additional patients (ALL_58, ALL_61, and ALL_257) with no evidence for a *ZNF384* rearrangement despite targeted screening (Fig. 3c, d). Unlike the *DUX4*-*IGH* group, patients with *ZNF384* rearrangements lacked differential expression of the *ZNF384* gene and its associated fusion partners (Additional file 3: Fig. S4B). 5′ RACE was performed to amplify the fusion transcripts without prior knowledge of the *ZNF384* fusion partner in these three patients. A novel fusion between the *ATP5C1* gene on chromosome 10 (5′ fusion partner) and *ZNF384* (3′ fusion partner) was detected in ALL_257 (Additional file 3: Fig. S5B). The RACE experiments were inconclusive for ALL_58 and ALL_61, and additional experiments will be needed to identify which, if any, *ZNF384* fusion gene is present in these patients.

Consistent with the previously reported Philadelphia-like signature associated with *PAX5*-*JAK2*, patient ALL_539 clustered together with the t(9;22)*BCR-ABL1* patients (Fig. 3e, f) [4, 33]. The remaining patients with other *PAX5* fusion genes clustered together.

### Differential DNA methylation and gene expression

To date, differential DNA methylation has not been comprehensively studied in the *DUX4*-*IGH*- and *ZNF384*-rearranged subgroups. We therefore highlight DMCs in combination with differentially expressed genes in patients with the *DUX4-IGH* and *ZNF384* rearrangements compared to patients with well-established ALL subtypes and normal CD19^+^ B cells.

**Fig. 4:**
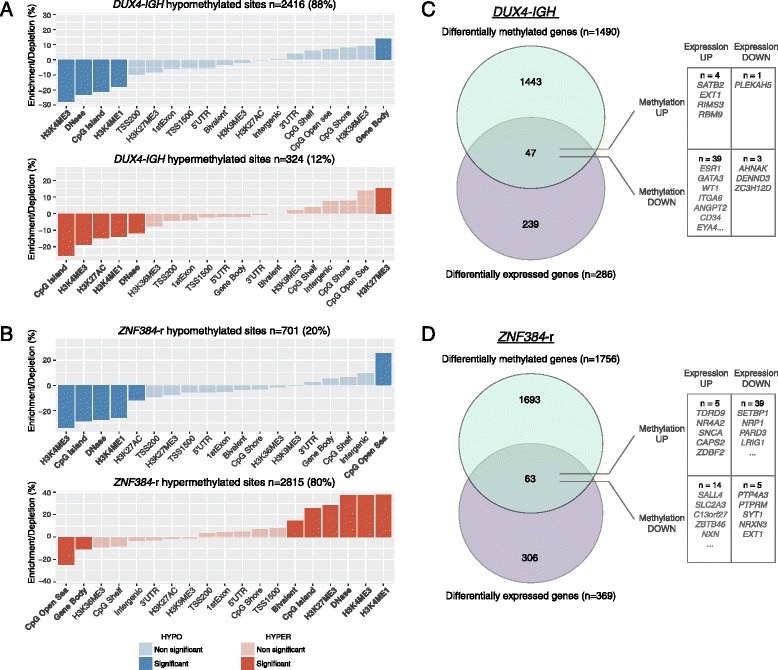
Functional genomic distribution of differentially methylated CpG sites (DMCs) in patients with *DUX4*-*IGH* and *ZNF384* rearrangements. **a**, **b** The enrichment and depletion of hypomethylated DMCs in *blue* and hypermethylated DMCs in *red* were determined in relation to CpG island context (island, shore, shelf, open sea), gene regions, and chromatin states in groups with **a** *DUX4*-*IGH* and **b** *ZNF384* rearrangements. The *bars* show the difference in proportion of the CpG sites annotated to each functionally annotated region between the 450 k array and the DMCs. The *colored bars* represent the annotations to which the DMCs significantly differ compared with the distribution of probes on the 450 k array (Bonferroni-corrected two-sided Fisher’s exact *P* value < 0.01 and absolute difference in proportion > 10). **c**, **d** Overlap between differentially methylated genes (*green*) and differentially expressed genes (*purple*) in patients with **c** *DUX4*-*IGH* and **d** *ZNF384* rearrangements. The overlapping genes with hypermethylated and hypomethylated DMCs and up- or downregulated gene expression are listed in the *right panel*

We detected 2740 and 3516 DMCs specific to the *DUX4-IGH*- and *ZNF384*-rearranged subgroups, respectively (Additional file 1: Table S8–S9). *DUX4*-*IGH* was characterized by widespread hypomethylation compared with normal B cells and the other ALL subtypes, whereas the group with *ZNF384* rearrangements was hypermethylated (Fig. 4, Additional file 1: Table S10). The DMCs were distributed across 245 and 192 genes unique to the *DUX4-IGH* and *ZNF384*-rearranged groups, respectively (Additional file 1: Table S10). While no enrichment to known pathways was observed, an enrichment of genes regulated by the transcription factor E2F1 was found in the *DUX4*-*IGH* subgroup (*p* = 8.2 × 10^− 5^). E2F1 is a member of the E2F family of transcription factors and acts as a potent transcriptional activator and master regulator of cell cycle progression.

To provide additional insights into possible functional implications of the subtype-specific DMCs, we examined their distribution across functional genomic regions defined by chromatin marks and DNaseI hypersensitive sites and in relation to CpG islands and gene-centric regions. The majority of the DMCs (88%) in *DUX4*-*IGH* were hypomethylated, enriched to gene bodies, and depleted in regions with open chromatin and high CG density (Fig. 4a). In contrast, the predominantly hypermethylated DMCs (80%) in the *ZNF384*-rearranged group were strongly enriched in CpG islands marked by bivalent chromatin marks (H3K4me3 and H3K27me3) and in open chromatin regions (Fig. 4b).

In order to investigate whether differential methylation is associated with gene expression, differential gene expression analysis was performed in the *DUX4*-*IGH*- and *ZNF384*-rearranged groups (Additional file 1: Table S11–S12). Approximately 3% of the genes with DMCs overlapped with differentially expressed genes corresponding to 47 and 63 overlapping genes in the *DUX4*-*IGH*- and *ZNF384*-rearranged groups, respectively (Fig. 4c, d, Additional file 1: Table S13). Most of the overlapping genes in the *DUX4*-*IGH* group showed an inverse correlation between methylation and gene expression (85%), with the majority of genes upregulated in *DUX4*-*IGH* compared to the other subtypes. Several of the overlapping genes (*n* = 9) were directly regulated or associated with the hypomethylated *ESR1* gene such as *GATA3*, *WT1*, and *ITGA6*. Alterations involving *ESR1* has mainly been described in breast cancer [34]. However, a study performed in non-hyperdiploid multiple myeloma proposed that *ESR1* contributes to cell cycle dysregulation, thus affecting the transcription of several downstream genes including *E2F1* [35]. Similarly, in the *ZNF384*-rearranged group, the majority of the genes showed an inverse correlation between methylation and gene expression (84%); however, the differentially expressed and methylated genes were downregulated compared to the other ALL subgroups. These include genes involved in hematological system development such as *SETBP1* and *NRP1* and putative tumor suppressor genes such as *PARD3* and *LRIG1* [36–38]. A notable exception is the overexpression of *SALL4* in the *ZNF384*-rearranged group, which has been described as an oncogene in leukemia [39, 40].

### Genetic alterations

To further characterize the subgroups with *DUX4*-*IGH* and *ZNF384* rearrangements, we utilized previously published targeted exome sequencing data including 872 cancer genes [29] and array-based copy number data derived from the HumanMethylation 450 k arrays from the same patients [19]. Targeted sequencing data was available for five out of the nine *DUX4*-*IGH* positive cases. Non-synonymous somatic mutations in the mutation hotspot p.G12 of the *NRAS* gene were found in all five *DUX4*-*IGH*-positive samples that were analyzed by targeted sequencing (Table 2). We screened for *NRAS* mutations in the RNA-sequencing reads confirming the presence of the five aforementioned *NRAS* mutations, but no additional *NRAS* mutations were detected in the remaining four patients. We detected *ERG* deletions, which is a common alteration in this subgroup [14, 15], in seven out of the nine *DUX4-IGH* patients (Additional file 3: Fig. S6). Interestingly, we also detected shared non-synonymous *PTPN11* mutations and chr7q deletions in the two patients with *TCF3*-*ZNF384* (Table 2). These mutations were confirmed in the RNA-sequencing data, and no additional *PTPN11* mutations were detected in the other patients with *ZNF384* rearrangements. Furthermore, Hirabayashi et al. reported that two out of six patients with *TCF3*-*ZNF384* harbored *PTPN11* mutations detected by whole exome sequencing or RNA sequencing; however, no additional copy number analysis was performed [13]. Together, these findings, albeit in a small sample set, suggest a common pattern related with the *TCF3*-*ZNF384* fusion gene.

**Table 2:** Genetic alterations in patients with *DUX4*-*IGH* and *ZNF384* rearrangements

Fusion gene	Sample	Mutated ALL driver genes^a^	Copy number alterations
*DUX4-IGH* fusion gene			
*DUX4*-*IGH*	ALL_176	na	*ERG* deletion
*DUX4*-*IGH*	ALL_218	na	Inconclusive
*DUX4*-*IGH*	ALL_312	*NRAS* (p.G12D)	*ERG* deletion
*DUX4*-*IGH*	ALL_371	*NRAS* (p.G12D)	*ERG* deletion
*DUX4*-*IGH*	ALL_390	na	*ERG* deletion
*DUX4*-*IGH*	ALL_392	na	*ERG* deletion
*DUX4*-*IGH*	ALL_501	*NRAS* (p.G12S), *KMT2D* (p.H3883fs)	*ERG* deletion
*DUX4*-*IGH*	ALL_546	*NRAS* (p.G12S)	*ERG* deletion
*DUX4-chr8q24.21-IGH*	ALL_205	*NRAS* (p.G12D)	Inconclusive
*ZNF384*-rearranged fusion genes			
*TCF3*-*ZNF384*	ALL_604	*PTPN11* (p.E76Q)	chr7q deletion
*TCF3*-*ZNF384*	ALL_622	*PTPN11* (p.T73I)	chr7q deletion
*TAF15*-*ZNF384*, *ZNF384*-*TAF15*	ALL_8	*NRAS* (p.Q61H)	–
*EP300*-*ZNF384*, *ZNF384*-*EP300*	ALL_52	na	–
*EP300*-*ZNF384*, *ZNF384*-*EP300*	ALL_613	na	–
*EP300*-*ZNF384*	ALL_693	na	–
*ATP5C1-ZNF384*	ALL_257	*CREBBP* (p.R1408C)	–

^a^Known ALL driver genes are reported in this table

*na* no targeted sequencing data available

### Clinical features

Recent studies have shown that *DUX4* rearrangements are associated with a favorable prognosis of pediatric ALL, whereas *ZNF384* rearrangements appear to be associated with an intermediate outcome. To assess the prognostic impact of recurrent fusion genes in our cohort, we determined the event-free survival (EFS) in the BCP-ALL subgroups (Additional file 3: Fig. S7). One relapse was observed in the nine patients with *DUX4*-*IGH*, which confirms previous reports of a generally favorable outcome associated with *DUX4* rearrangements [9, 14, 15]. Inferior prognosis has been associated with *TCF3*-*ZNF384*, while favorable prognosis has been associated with the *EP300-ZNF384* fusion gene [13]. In the present study, no relapses were observed in patients with *EP300-ZNF384*. Relapses were observed in patients with all of the other fusion gene partners (*TCF3*-*ZNF384* (ALL_622), *TAF15*-*ZNF384* (ALL_8), and *ATP5C1-ZNF384* (ALL_257)).

## Discussion

In our study, we screened the whole transcriptome of 134 pediatric ALL patients by RNA sequencing to comprehensively identify expressed fusion genes. Our results highlight recurrent fusion gene families, comprising recurrent fusion genes and promiscuous genes that form fusion genes with several partners in the heterogeneous BCP-ALL “other” group, and add to the repertoire of recurrent fusion events in pediatric ALL.

Accurate identification of fusion genes requires a balance between increasing sensitivity in combination with reduction of false positives by filtering and validation experiments. Even after computational filtering, we performed PCR and Sanger sequencing to validate each unique fusion gene in comparison with CD19^+^ B cells and CD3^+^ T cells from healthy blood donors to ascertain that all identified putative fusion gene was expressed exclusively in ALL cells. This approach excluded 2075 of the putative fusion genes called by FusionCatcher [23], and thus, we identified an average of 1.8 fusion genes per sample validated with high confidence in BCP-ALL and 1.0 fusion genes per sample in T-ALL.

The most significant recent findings in terms of fusion genes in pediatric ALL are the fusion genes involving *DUX4*, *ZNF384*, and *MEF2D* [9, 10, 14]. We detected *DUX4-IGH* in 21% of our BCP-ALL “other” patients and various *ZNF384* rearrangements in 17% of our BCP-ALL “other” patients. The children in these groups were older at diagnosis (median 9.3 years in *DUX4*-*IGH* and 10.7 in *ZNF384*-rearranged vs 4.5 years in BCP-ALL) and exhibited similar clinical outcome as in previous studies [9, 14, 15, 41–43]. Because of these consistent findings, *DUX4-IGH* should be considered as a potential favorable clinical marker in BCP-ALL and evaluated together with other prognostic factors in larger ALL cohorts.

To date, four studies have screened for somatic mutations in *DUX4*-*IGH*- and *ZNF384*-rearranged cases [12–15]. *NRAS* mutations are present in approximately 20% of BCP-ALL samples [29] and are enriched in the HeH subtype [44]. *NRAS* mutations have been reported in 13–26% of *DUX4*-rearranged cases, yet we found them in > 50% of cases. Our results, although based on a small number of samples, indicate that the frequency of *NRAS* mutation may in fact be higher in this subgroup. Both of the *TCF3*-*ZNF384* patients in the present study harbored somatic *PTPN11* mutations. In the few *ZNF384*-rearranged cases described in the literature, *PTPN11* mutations have only been observed in *ZNF384*-rearranged cases when they have the *TCF3* partner gene [12, 13]. These observations, however, are based on a small number of samples, and larger cohorts should be investigated to determine the actual mutational frequencies and if the mutations have any prognostic impact.

*MEF2D* fusion genes have recently been reported to comprise a new biological subtype of BCP-ALL with an overall frequency of 4–7% but are predominantly found in young adults [9, 10, 45, 46]. We did not identify any *MEF2D* fusion genes, despite performing additional targeted screening (Additional file 1: Table S4). Notably, Lilljebjörn et al. detected one patient with a *MEF2D* fusion gene in an independent cohort of 195 Swedish pediatric BCP-ALL patients [14]. The younger average age of the patients in Lilljebjörn et al. and the present study (7.1 and 5 years, respectively) most likely explains why so few *MEF2D* fusion genes were detected in the two Nordic cohorts.

Fusion genes can affect cellular functions through several means including formation of constitutively active chimeric proteins or by creating dominant negative proteins that inhibit normal protein function, which in turn could lead to the malignant transformation into cancer cells [47]. Fifty-six percent of the fusion genes detected in the current study were in-frame and the subtype-defining fusion genes are typically in-frame (*ETV6*-*RUNX1*, *BCR*-*ABL1*, and *KMT2A* rearrangements; *TCF3*-*PBX1*, *DUX4*-*IGH*, and *ZNF384* rearrangements). Even reciprocal fusion genes arising from balanced breakpoints, when expressed, are typically in-frame. A notable exception to this pattern is the t(12;21)-like fusion genes consisting of *RUNX1*-*ASXL1*, *CBX3*-*ETV6*, and *ETV6*-*AK125726*, which were all out-of-frame. Despite this, the patients harboring t(12;21)-like out-of-frame fusion genes, display highly similar DNA methylation patterns as the t(12;21)-positive cases, as we have previously described [8] and similar gene expression patterns as showed in the present study and by others [14]. T(12;21)-like fusion genes also infer similar favorable clinical outcomes as *ETV6*-*RUNX1*, which raises the interesting point whether these fusion genes could be used for treatment stratification purposes. We also identified fusion genes in 11/42 HeH patients, which is more common than previously observed [14]. With the exception of *ARHGEF6*–*ORC4* and *P2RY8*-*CRLF2*, all the other fusion genes detected in HeH were out-of-frame and most were intra-chromosomal.

## Conclusion

Expressed fusion genes are a recognized form of driver mutations in ALL, and the insights gained from our study will help to shape our understanding of ALL pathogenesis and the heterogeneity between patients. Moreover, studies like these offer the possibility to identify targetable fusion genes as has been done for tyrosine kinase-related fusion genes [11, 48, 49]. Considering the decrease in sequencing costs coupled with recent advances in deep-sequencing technology, unbiased and detailed screening of fusion genes is now feasible. The next challenge will be to identify the downstream targets and cellular processes that are affected by fusion genes, which in turn may lead to refined stratification of patients for existing therapies and the discovery of new therapies for ALL.

## Additional files

Additional file 1: Supplementary Tables S1-S1﻿3. (XLSX 1165 kb)

Additional file 2: Additional materials and methods. (DOCX 29 kb)

Additional file 3: Supp﻿l﻿ementary Figures S1-S7﻿. (PDF 2992 kb)

## Abbreviations

ALL: Acute lymphoblastic leukemia
BCP-ALL: B cell precursor acute lymphoblastic leukemia
CNA: Copy number analysis
DMC: Differentially methylated CpG site
EFS: Event-free survival
FPKM: Fragments per kilobase per million mapped reads
HeH: High hyperdiploid
NOPHO: Nordic Society of Pediatric Hematology and Oncology
RACE: Rapid amplification of cDNA ends
SNVs: Single nucleotide variants
T-ALL: T cell acute lymphoblastic leukemia
