# Supplementary material for: Transcriptome sequencing in pediatric acute lymphoblastic leukemia identifies fusion genes associated with distinct DNA methylation profiles
Source: J Hematol Oncol. 2017 Aug 14;10:148. doi: 10.1186/s13045-017-0515-y (PMC5557398; doi:10.1186/s13045-017-0515-y)
Supplement: Supplementary file 2 — Additional materials and methods. (DOCX 29 kb) [file 13045_2017_515_MOESM2_ESM.docx]

# Supplementary materials and methods

## Patient samples

Bone marrow aspirates or peripheral blood samples were collected at diagnosis from 134 pediatric ALL patients enrolled between 1996 and 2010 on the Nordic Society of Pediatric Hematology and Oncology (NOPHO) ALL-92, ALL-2000, or ALL-2008 protocols, EsPh-ALL, Interfant-99 or Interfant-06 protocols. The high hyperdiploidy (HeH) subtype was assigned to patients with 51-67 chromosomes per cell. FISH and/or RT-PCR was used to screen for the translocations t(12;21)(p13;q22)*ETV6*-*RUNX1* and t(9;22)(q34;q11)*BCR-ABL1*. FISH or Southern blot analysis were used to identify 11q23/*MLL* rearrangements.

Clinical follow-up data was collected from the Nordic Society of Pediatric Hematology and Oncology (NOPHO) registry with a median follow-up time of 12 years (range 8 to 20 years).

Normal CD19+ B cells (n=5) and CD3+ T cells (n=5) isolated from peripheral blood mononuclear cells from five healthy Swedish blood donors using positive selection (CD19 Microbeads #120-050-301 and CD3 Microbeads #130-050-101) and MACS magnetic cell separation reagents (Miltenyi Biotec, Bergisch Gladbach, Germany), served as a reference.

## RNA extraction

RNA was extracted from 2-15 million cells after Ficoll gradient separation (ALL samples) or positive selection (control samples) using reagents from the AllPrep DNA/RNA Kit including a DNase treatment step (Qiagen). RNA was quantified with the Broad Range RNA kit on a Qubit instrument (Life Technologies) and the RNA quality was assessed using a Bioanalyzer (Agilent). All RNA samples included in the study had RNA integrity (RIN) values >7.

## RNA-sequencing and computational analysis

Ribosomal RNA (rRNA) was depleted from 1 µg of total RNA using the Ribo-Zero Magnetic Gold Kit (Epicentre) prior to library preparation with ScriptSeq V2 Kit according to the manufacturer’s instructions (Epicentre). Libraries were quality controlled using the High Sensitivity DNA analysis chip on a Bioanalyzer (Agilent) and quantified by quantitative real-time PCR using the KAPA Library Quantification Kit for the Illumina Platform (Kapa Biosystems). Libraries were sequenced on an Illumina Hiseq2000/2500 instrument, 50bp paired-end, with the exception of one sample (ALL_707) that was sequenced on a MiSeq instrument, 83bp paired-end. Sequencing was performed using TruSeq SBS Chemistry v3 (HiSeq) or MiSeq Reagent Kit v3 according to the manufacturer’s protocols. Base calling of data from the Illumina systems was performed on the instrument and the resulting BCL files were filtered, de-multiplexed, and converted to FASTQ format using CASAVA v1.8.2. Raw sequence reads were adaptor-trimmed using Cutadapt 1.2.1 ([1](#_ENREF_1)) and mapped to the genome (human_g1k_v37) using Tophat 2 (2.0.4) ([2](#_ENREF_2)) as implemented in the “Piper” pipeline for RNA-sequencing data analysis (https://github.com/johandahlberg/piper/tree/rna_all_piper_version). The following parameters were used for Tophat 2: --library-type fr-secondstrand --GTF [Ensembl GRCh37 release 66] -p 8 --keep-fasta-order. An average of 46 million read pairs per sample was obtained. Quality control of RNA-sequencing data was performed with RNA-SeQC. Over 90% of the reads were required to be in the correct orientation (strand specificity over 90%) to pass quality control (**Additional file 1: Table S2**) ([3](#_ENREF_3)).

## Fusion gene detection and validation

FusionCatcher 0.99.4a beta was used to detect both novel and known fusion chimeras using the “--highly-sensitive” mode ([4](#_ENREF_4)). To reduce the number of false-positives, we applied a stringent filtering process of the raw fusion genes detected by FusionCatcher (**Additional file 3: Figure S1B**). These measures consisted of removing blacklisted fusion genes based on reference data provided by FusionCatcher, removing fusion genes based on common mapping read (fusion-supporting read map to multiple places and indicative of sequence homology), if the 5’ or the 3’-fusion gene partner mapped to several other genes (highly promiscuous gene) or if less than three sequencing reads were found to support a detected chimeric transcript (**Additional file 1: Table S3**). Fusion genes that were identified in the RNA-sequencing data from normal B/T-cell samples were filtered out of the ALL-fusion calls in order to enrich for cancer-specific fusions. Genes previously described to be involved in ALL-related fusion events were retained throughout all filtering steps. Fusion genes that were filtered away are listed in **Additional file 1: Table S3**.

In a second step we performed a targeted screening for 22 well-established and recognized fusion genes previously reported in BCP-ALL and T-ALL, including the canonical fusion genes associated with t(12;21), t(9;22) and 11q23/*MLL* subtypes (**Additional file 1: Table S4, Additional file 3: Figure S1A**). Aligned fusion supporting read pairs were counted and normalized by the total number of sequencing read pairs in the sample. A fusion supporting read pair is defined as one read in a read pair mapping to the 5’ fusion gene partner and the other read in the same read pair mapping to the 3’ fusion gene partner. This process was implemented as a python script available here <https://github.com/Molmed/Marincevic-2017>. Parsing of bam files was carried out using PySam (<https://github.com/pysam-developers/pysam>) ([5](#_ENREF_5)).

Candidate fusion genes detected by FusionCatcher were validated in a two-step approach that consisted of visual inspection of the aligned reads that supported a fusion transcript using the Integrative Genomics Viewer (IGV) ([6](#_ENREF_6)), followed by experimental validation by PCR and Sanger sequencing (**Additional file 3: Figure S1B-C**).

Primers spanning the fusion junction were designed using the Primer3 software (**Additional file 1: Table S5**). Starting with 250 ng RNA, cDNA was synthesized using the SuperScript III First Strand cDNA synthesis kit (Life Technologies). RNA from normal CD19+ B cells or CD3+ T cells was included as a negative control to determine whether the fusion gene was specific for the leukemic samples. Phusion Hot Start II DNA Polymerase kit (Thermo Scientific) was used for PCR with 30 ng cDNA as input and amplicons were checked for correct size using gel electrophoresis. PCR products were treated with Exo-Sap followed by Sanger sequencing using AB BigDye Terminator v3.1 chemistry and processed on the ABI3730XL DNA Analyzer. Sequences were extracted using Sequence Scanner Software (Applied Biosystems) and aligned to the human reference genome using BLAT. Fusion genes concurrently identified in the normal CD19+ B and CD3+ T cells and in the leukemic samples by PCR and Sanger sequencing were excluded from further analysis to enrich for cancer-specific fusion transcripts. Validation by Sanger sequencing was not performed for well-established fusion genes including *ETV6-RUNX1,* *BCR-ABL1*, *P2RY8-CRLF2*, *DUX4-IGH* and *STIL-TAL1*.

## Data analysis and visualization

Data were visualized and plotted using R version 3.2.3 (R Core Team 2015) with the ggplot2 ([7](#_ENREF_7)) reshape2 ([8](#_ENREF_8)) and dplyr libraries (https://github.com/hadley/dplyr). All scripts used to manage the detection of fusion genes used in this article are available at: <https://github.com/Molmed/Nordlund-Dahlberg-2014>. Event-free survival was estimated using the Kaplan-Meier method. All groups were compared using the log-rank test. An event was defined as resistant disease, induction failure, relapse, death in remission or second malignant neoplasm.

# References

1. Martin M. Cutadapt removes adapter sequences from high-throughput sequencing reads. EMBnetjournal doi: <http://dxdoiorg/1014806/ej171200>. 2011.

2. Kim D, Pertea G, Trapnell C, Pimentel H, Kelley R, Salzberg SL. TopHat2: accurate alignment of transcriptomes in the presence of insertions, deletions and gene fusions. Genome biology. 2013;14(4):R36. PubMed PMID: 23618408. Pubmed Central PMCID: 4053844.

3. DeLuca DS, Levin JZ, Sivachenko A, Fennell T, Nazaire MD, Williams C, et al. RNA-SeQC: RNA-seq metrics for quality control and process optimization. Bioinformatics. 2012 Jun 1;28(11):1530-2. PubMed PMID: 22539670. Pubmed Central PMCID: 3356847.

4. Nicorici DS, M.; Edgren, H.; Kangaspeska, S.; Murumagi, A.; Kallioniemi, O.; Virtanen, S.;Kilkku, O. FusionCatcher - a tool for finding somatic fusion genes in paired-end RNA-sequencing data. bioRxiv doi: <http://dxdoiorg/101101/011650>. 2014.

5. Li H, Handsaker B, Wysoker A, Fennell T, Ruan J, Homer N, et al. The Sequence Alignment/Map format and SAMtools. Bioinformatics. 2009 Aug 15;25(16):2078-9. PubMed PMID: 19505943. Pubmed Central PMCID: 2723002.

6. Robinson JT, Thorvaldsdottir H, Winckler W, Guttman M, Lander ES, Getz G, et al. Integrative genomics viewer. Nature biotechnology. 2011 Jan;29(1):24-6. PubMed PMID: 21221095. Pubmed Central PMCID: 3346182.

7. Wickham H. ggplot2 - Elegant Graphics for Data Analysis: Springer-Verlag New York; 2009.

8. Wickham H. Reshaping data with the reshape package. J Stat Softw. 2007 Oct;21(12):1-20. PubMed PMID: WOS:000252429400001. English.
