# Supplementary material for: Transcriptome sequencing in pediatric acute lymphoblastic leukemia identifies fusion genes associated with distinct DNA methylation profiles
Source: J Hematol Oncol. 2017 Aug 14;10:148. doi: 10.1186/s13045-017-0515-y (PMC5557398; doi:10.1186/s13045-017-0515-y)
Supplement: Supplementary file 3 — Supp﻿l﻿ementary Figures S1-S7﻿. (PDF 2992 kb) [file 13045_2017_515_MOESM3_ESM.pdf]

Supplementary Figure 1

Suppl 1A. Combined approach for fusion gene detection

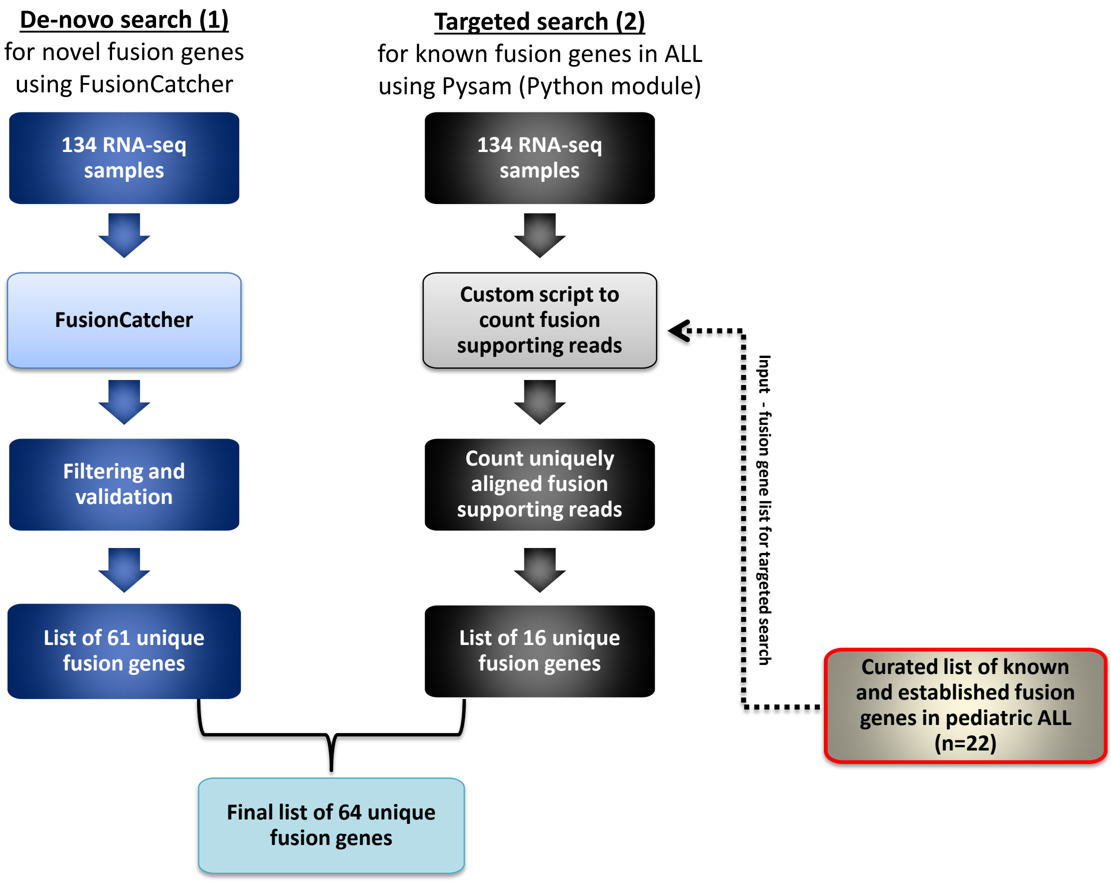

Suppl 1B. Filtering fusion genes called by FusionCatcher

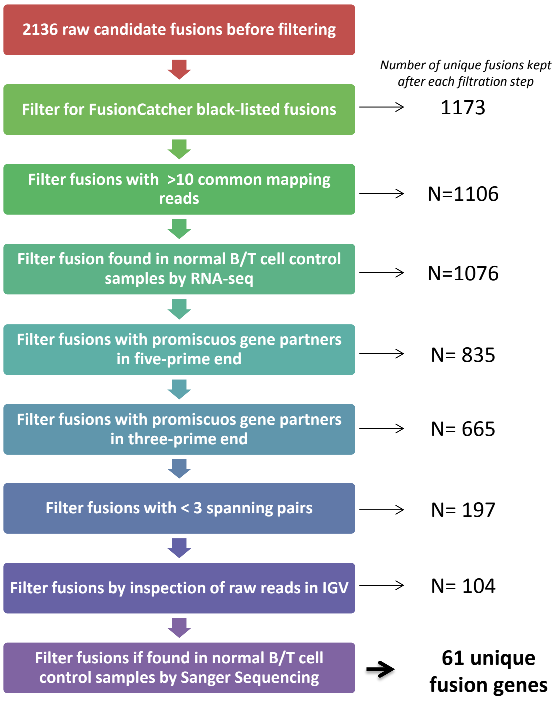

Supplementary Figure 1C

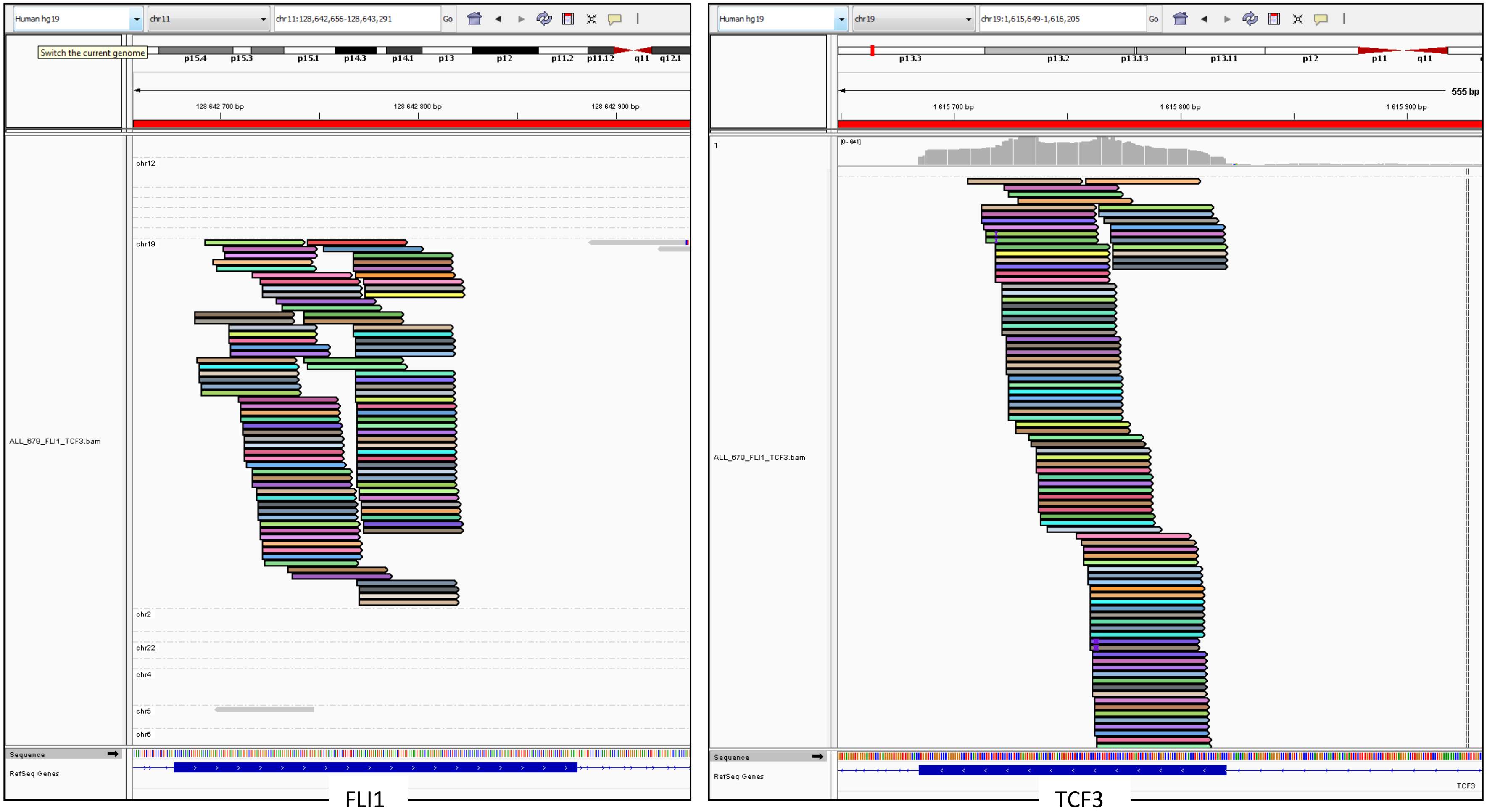

Fusion supporting reads for a true positive fusion gene (*FLI1-TCF3*) visualized in IGV. Paired-end RNA-sequencing reads where one mate map to *FLI1* (chr11) (left panel) and the other mate map to *TCF3* (chr19) (right panel) are highlighted with colors.

Supplementary Figure 1C

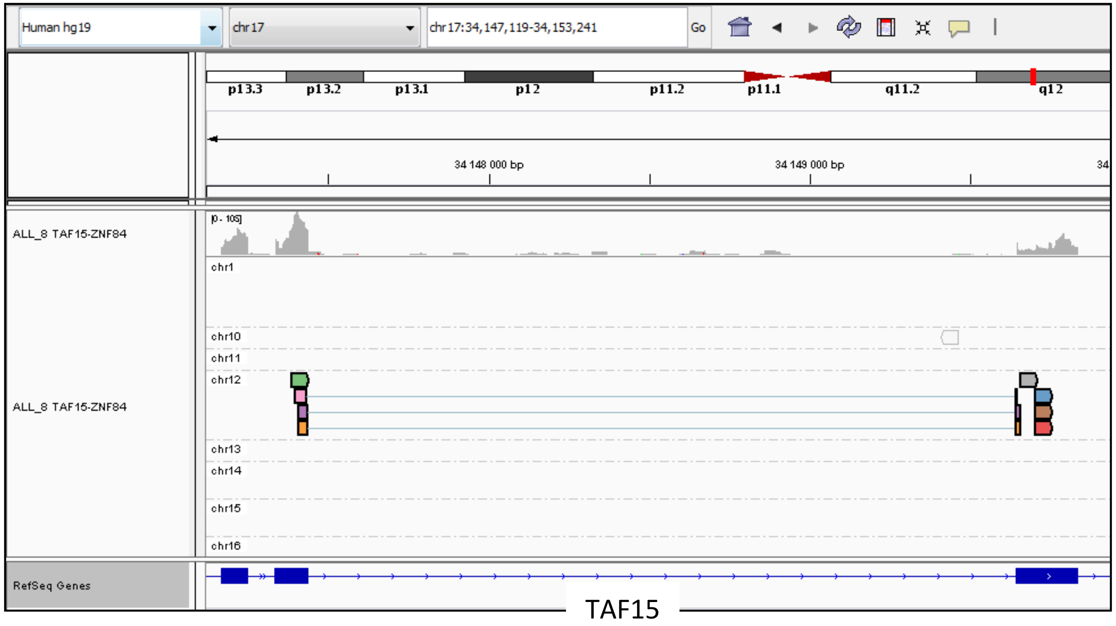

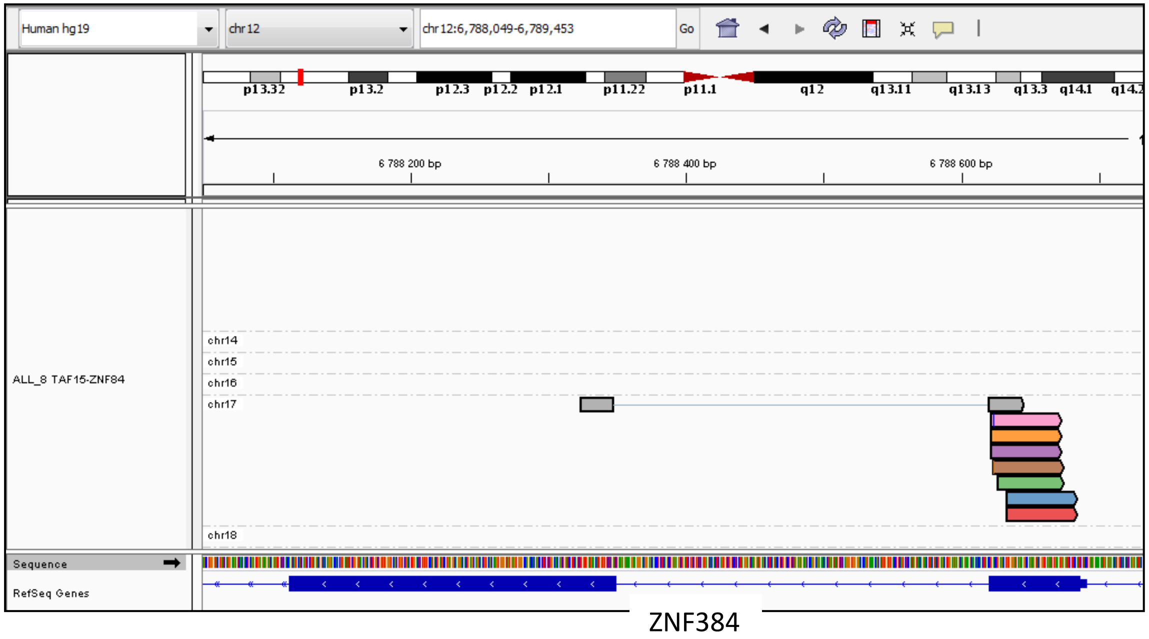

Fusion supporting reads for a true positive fusion gene (*TAF15-ZNF384*) visualized in IGV. Paired-end RNA-sequencing reads where one mate map to *TAF15* (chr17) (upper panel) and the other mate map to *ZNF384* (chr12) (lower panel) are highlighted with colors

Supplementary Figure 1C

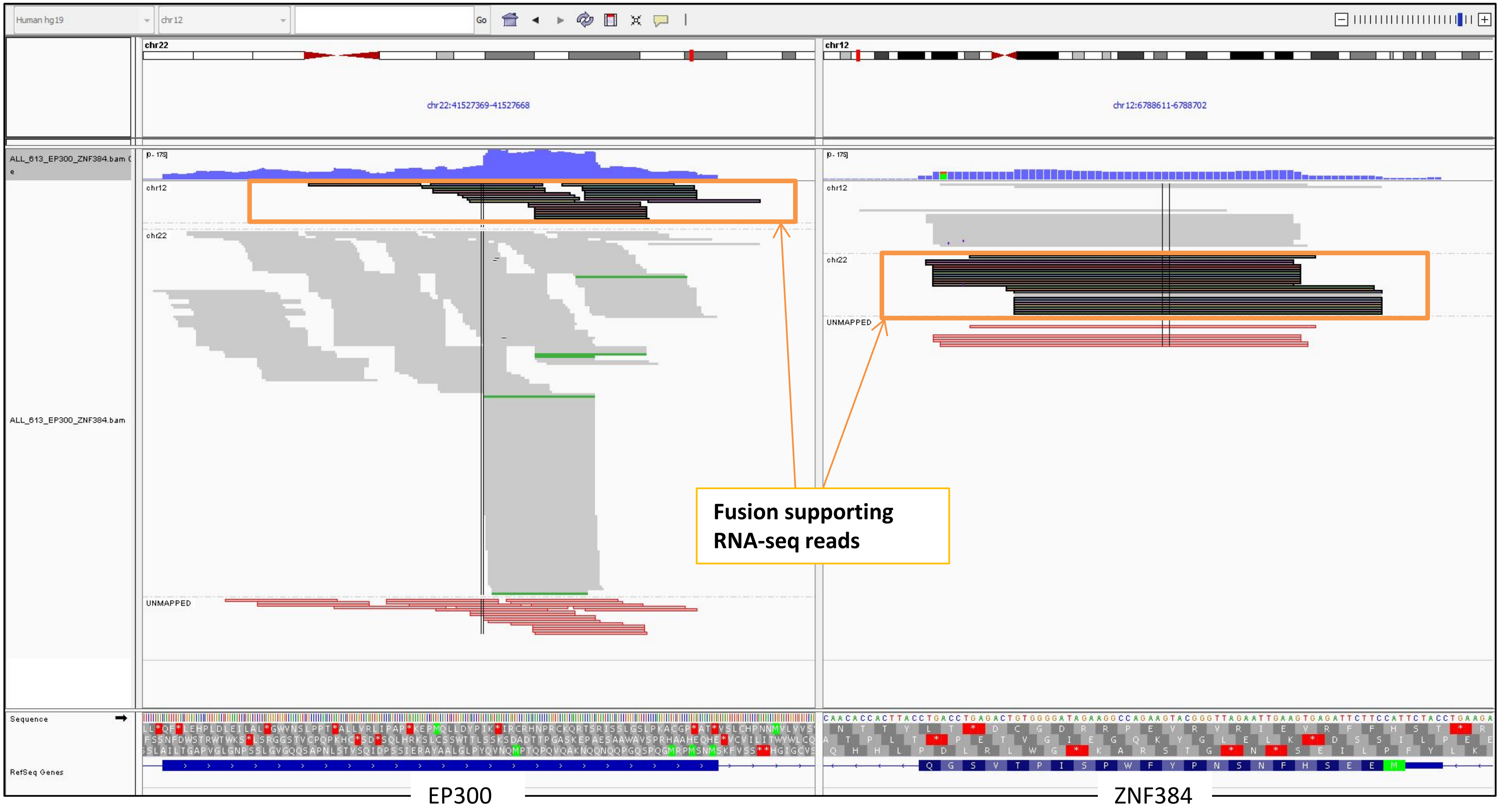

Fusion supporting reads for a true positive fusion gene (*EP300-ZNF384*) visualized in IGV. Paired-end RNA-sequencing reads where one mate map to *EP300* (chr22) (left panel) and the other mate map to *ZNF384* (chr12) (right panel) are highlighted with colors.

Supplementary Figure 1C

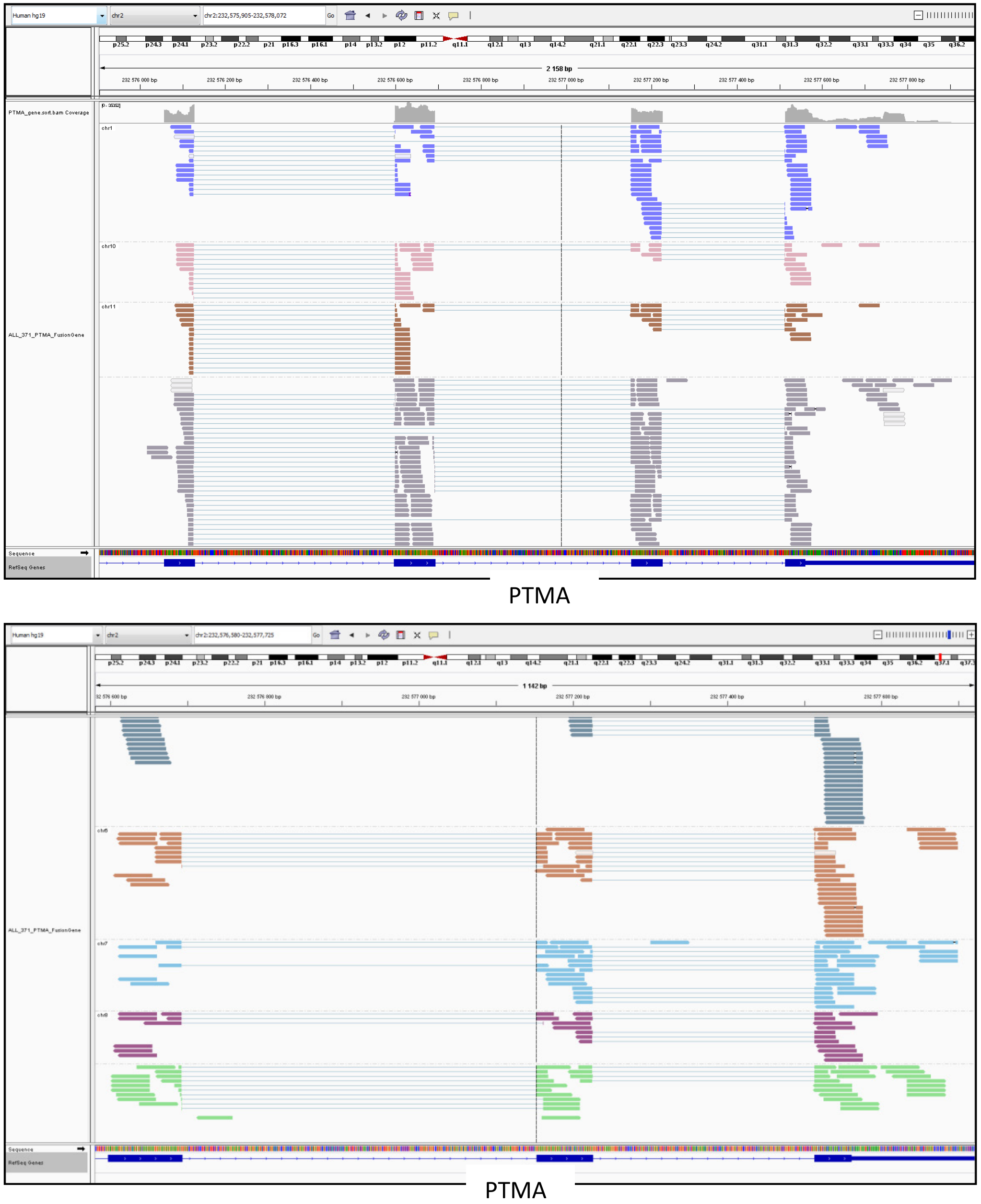

False positive fusion gene involving *PTMA* visualized in IGV. Paired end RNA-sequencing reads where one mate map to *PTMA* (chr2) and the other mate map to several different chromosomes illustrated by the different colors. Upper panel shows paired reads on *PTMA* where the mate map to chr 1, chr 10 and chr 11. Lower panel show paired reads mapping to chr 5, chr 6, chr 7, chr 8 and chr 9. Fusion genes involving *PTMA* or other promiscuous genes (Additional File1, Table S3B) were filtered away.

Supplementary Figure 1C

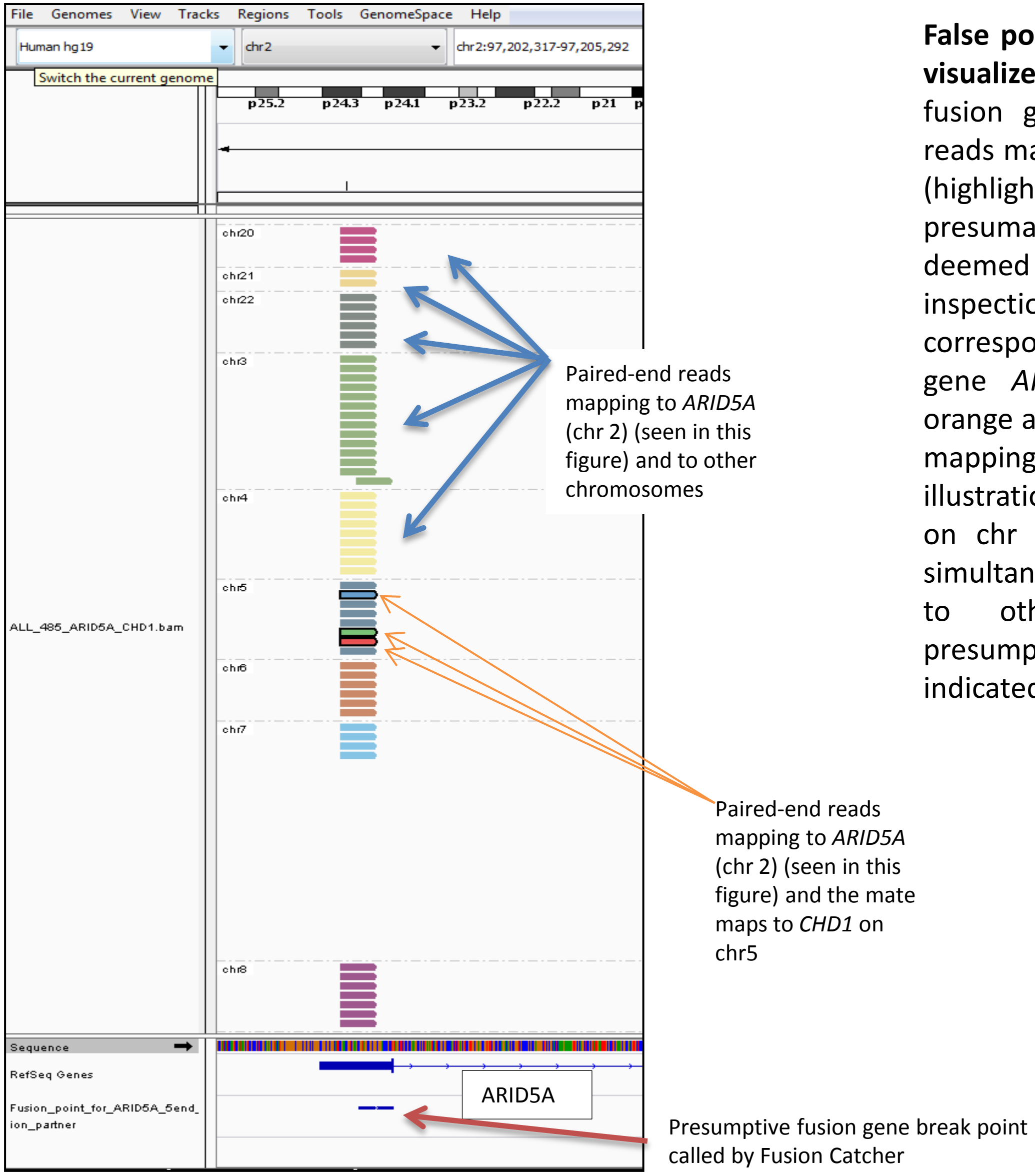

False positive fusion gene *ARID5A-CHD1* visualized in IGV. Inspection of candidate fusion genes in IGV where paired-end reads map to several other chromosomes (highlighted with different colors) at the presumable fusion gene breakpoint were deemed as false positives by IGV inspection. In this illustration, reads corresponding to the candidate fusion gene *ARID5A-CHD1* are indicted with orange arrows and represent paired reads mapping to *ARID5A* (chr2) (seen in this illustration) and the mate maps to *CHD1* on chr 5. A selection of paired reads simultaneously mapping to *ARID5A* and to other chromosomes at the presumptive fusion gene breakpoint are indicated with blue arrows

Supplementary Figure 1C

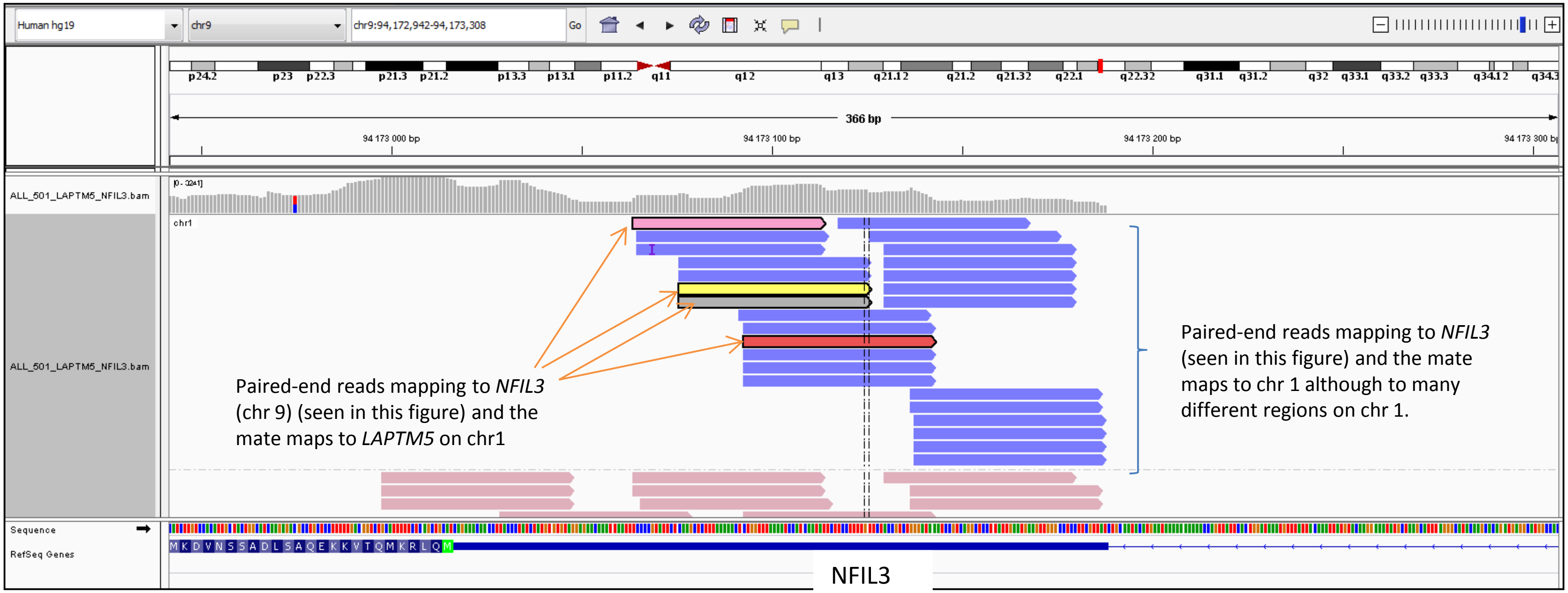

False positive fusion gene *LAPTM5-NFIL3* visualized in IGV. Inspection of candidate fusion genes in IGV where paired-end reads map to the expected chromosome (in this case chr 1) although to several different regions across the whole chromosome, were deemed as false positives by IGV. In this illustration, reads corresponding to the candidate fusion gene *LAPTM5-NFIL3* are highlighted with color and reads simultaneously mapping to *NFIL3* and to other regions across the whole chr1 are marked in blue.

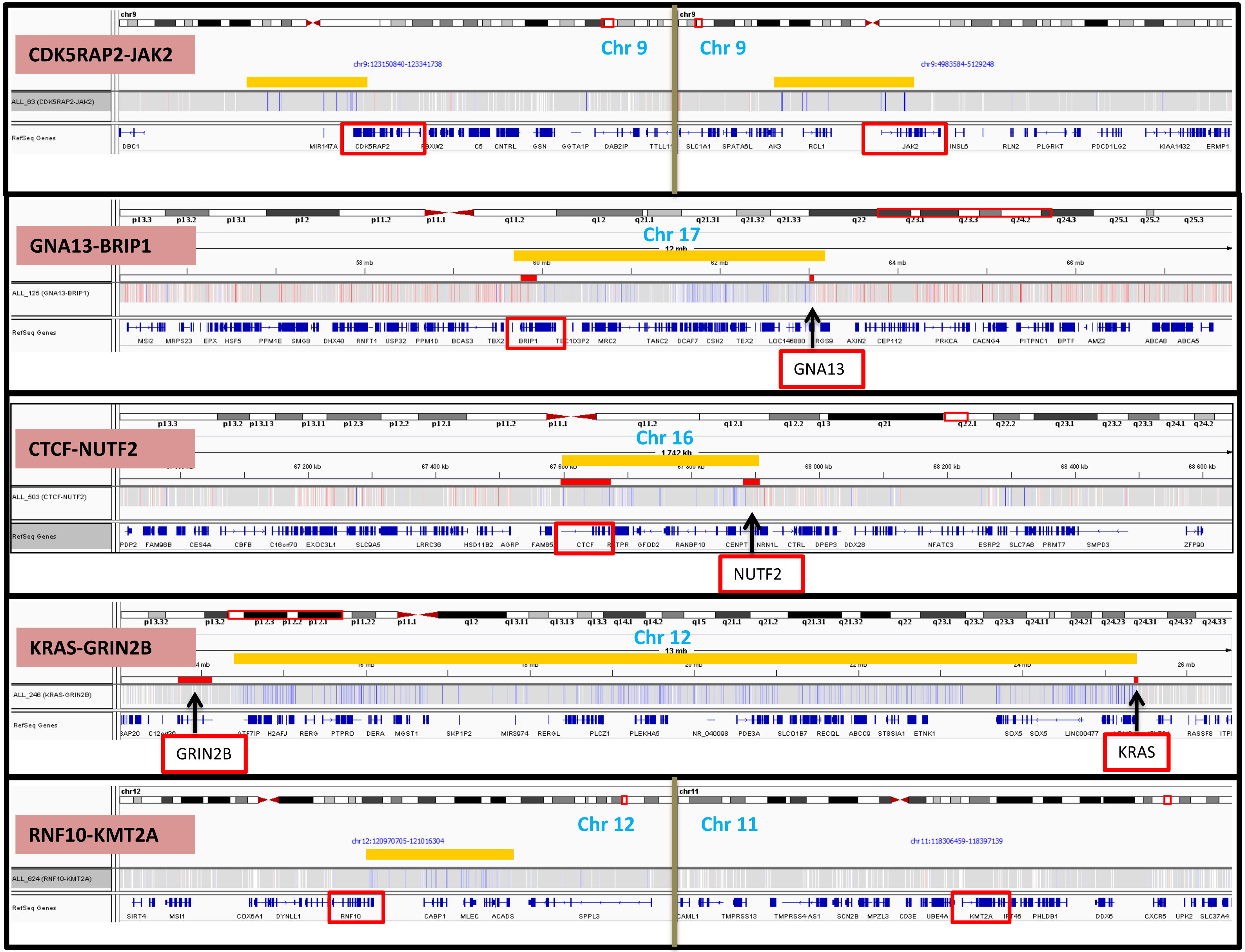

Supplementary Figure 2

Supplementary Figure 2

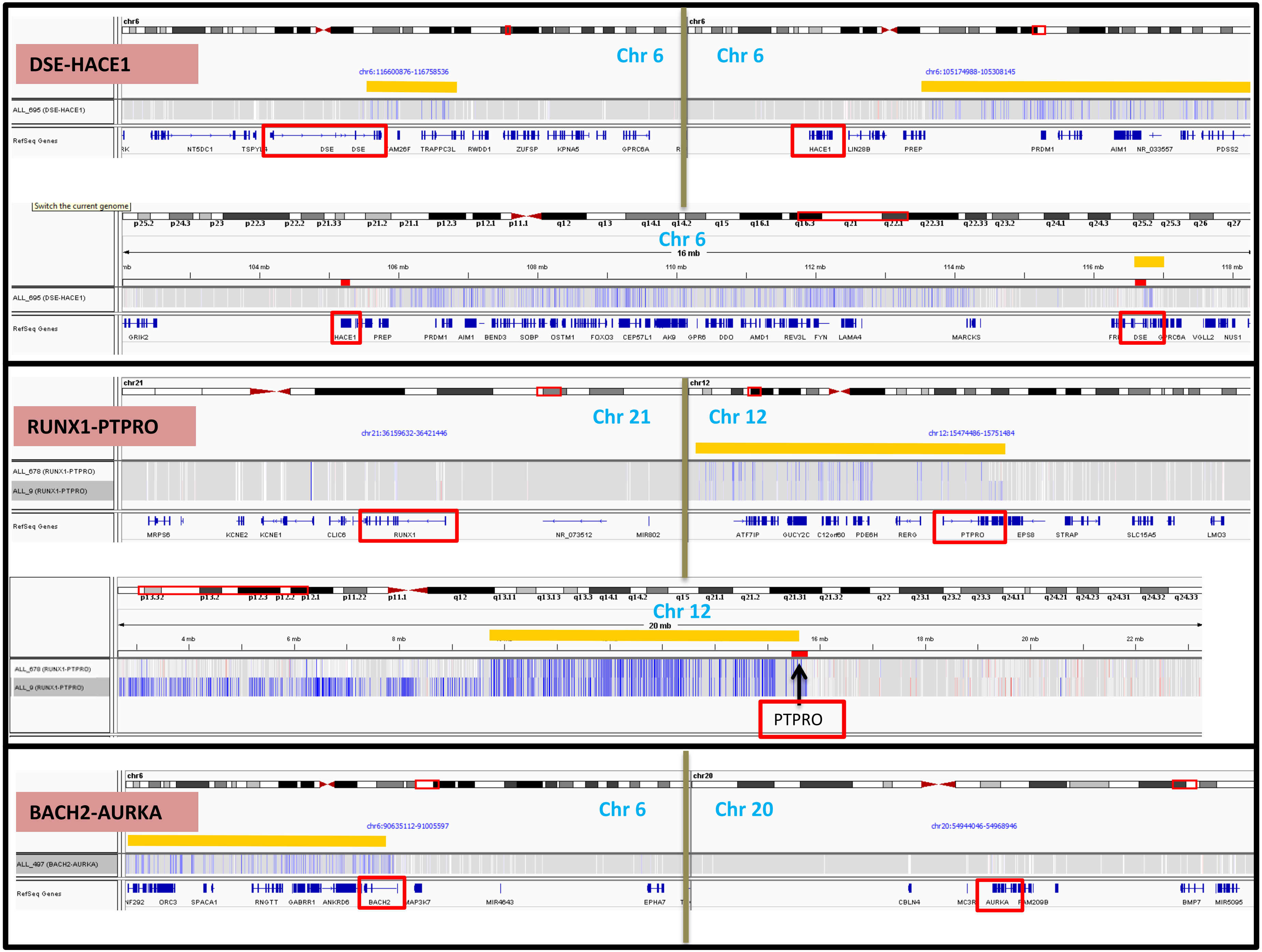

Supplementary Figure 2

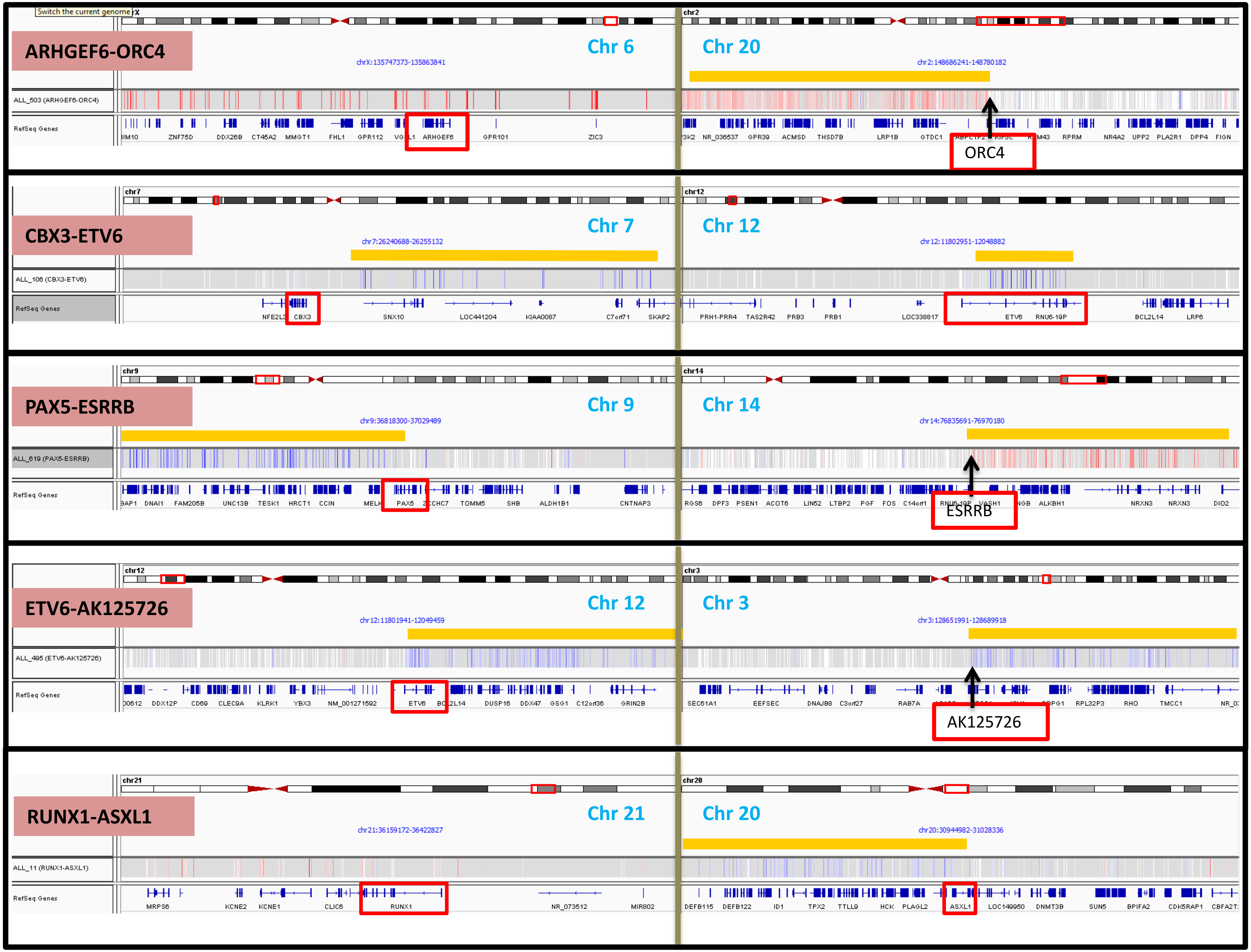

Supplementary Figure 2

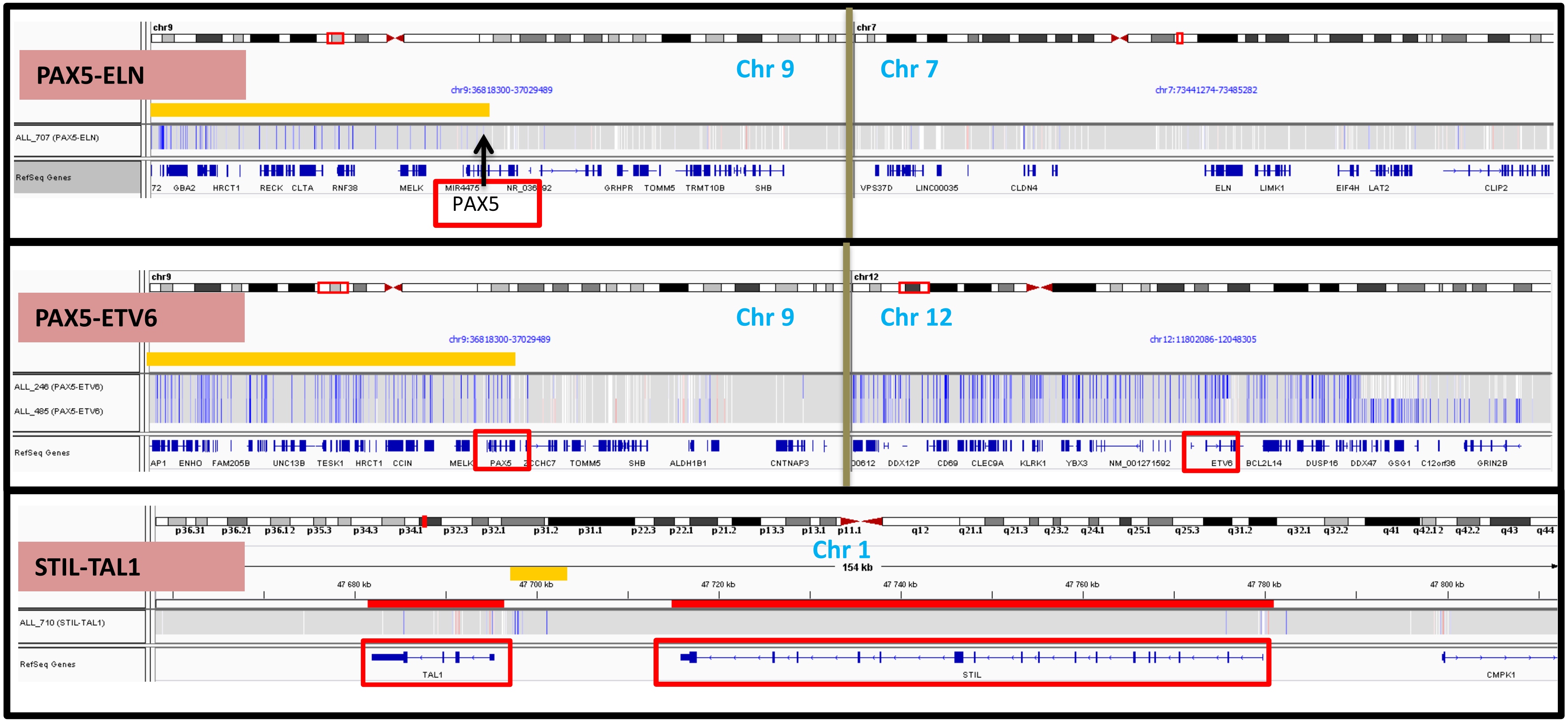

Supplementary Figure 2: Copy number alterations occurring in or in proximity of genes involved in a fusion gene. Copy number alterations (CNA) were detected using probe intensity levels from the Infinium HumanMethylation450 BeadChip. LogR ratios were calculated from the probe intensity levels and used to screen for CNA using the Integrative Genomics Viewer (IGV). Deletions or amplifications that show evidence for a copy number alteration, within or in proximity of a gene involved in a fusion gene, is highlighted with a yellow bar. Probes that are deleted are blue colored and probes that are amplified are red colored and represented by the lines in the grey panel. Each panel represents one fusion gene and is enclosed with a black frame. Panels with a split view for simultaneous visualization of two different chromosomes are marked with a brown line with respective chromosome region noted on each side in blue color. Fusion gene partners are highlighted with a red box.

Supplementary Figure 3

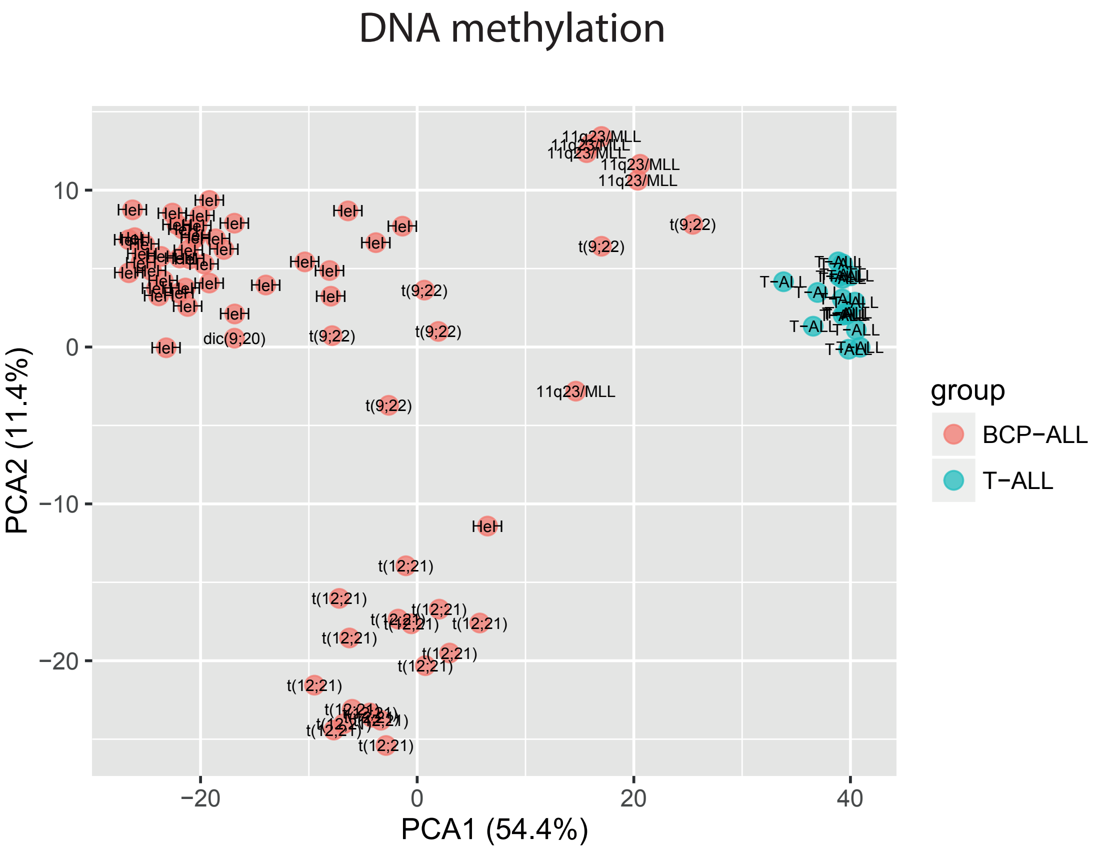

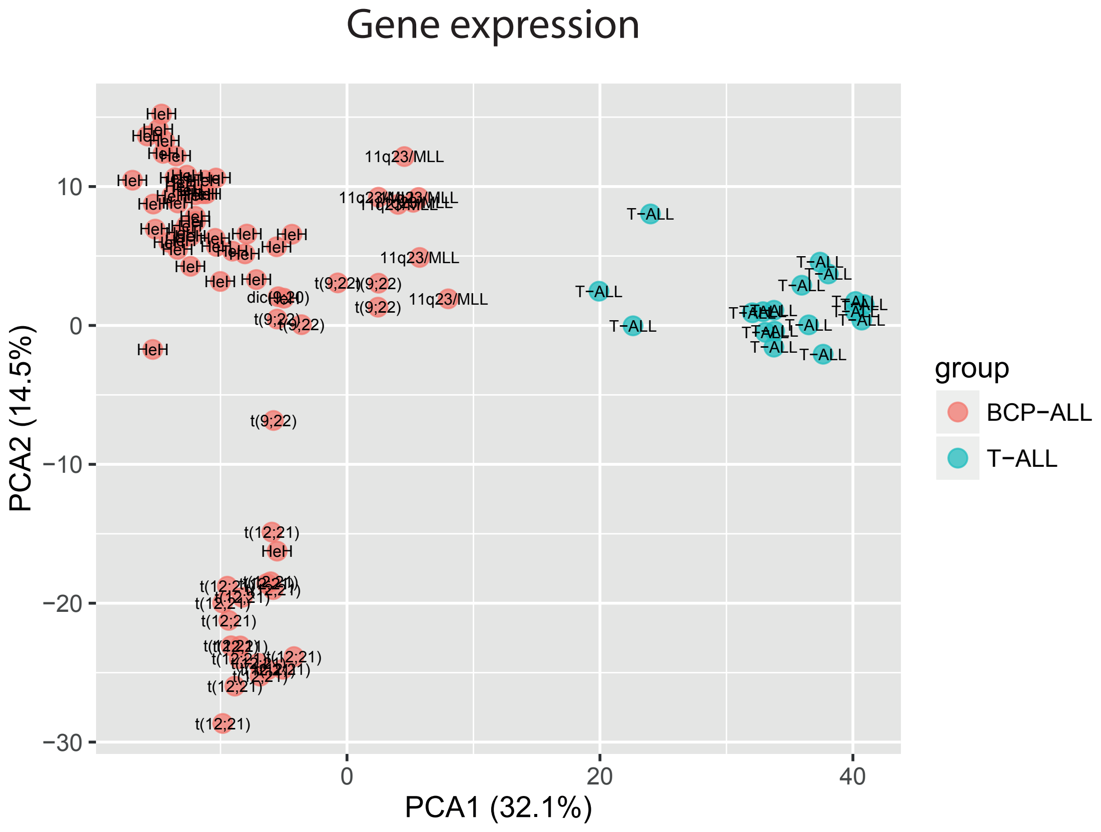

Supplementary Figure 3: Principal component analysis using genome-wide DNA-methylation and gene expression patterns of ALL patients. ALL patient samples clustered based on the immunophenotypes BCP-ALL or T-ALL.

Supplementary Figure 4

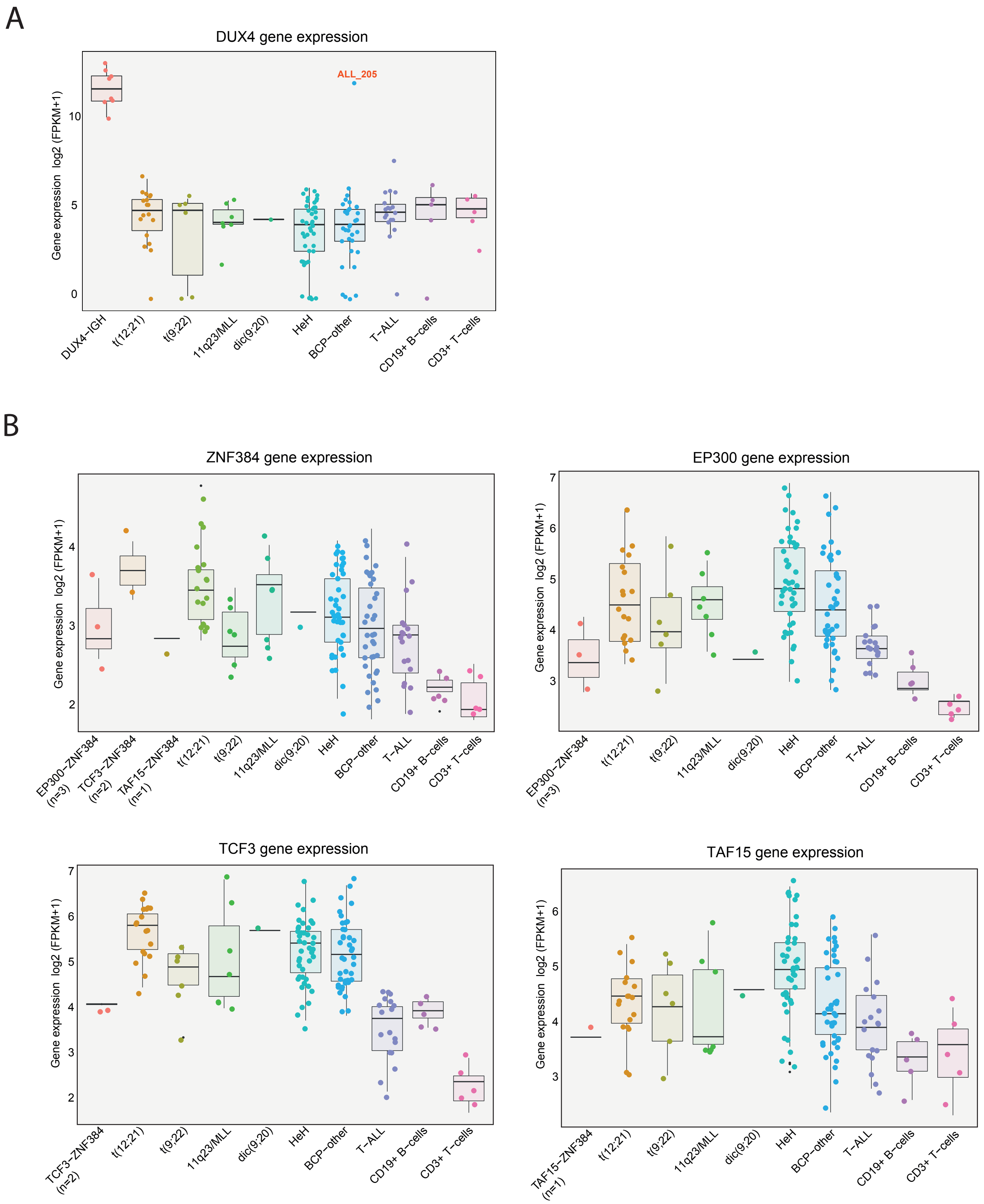

Supplementary Figure 4: Gene expression levels for fusion gene partners in DUX4-IGH and ZNF384-rearranged patients. (A) High DUX4 expression was confirmed in all eight DUX4-IGH patients compared to the other subtypes. One BCP-ALL "other" patient (ALL_205 - highlighted in red color) displayed high DUX4 expression levels and clustered together with the other DUX4-IGH positive samples.

(B) Expression levels of ZNF384 and fusion gene partners EP300, TCF3 and TAF15 revealed no difference in expression between the known ZNF384-rearranged cases and the other BCP-ALL subtypes and controls. Only one of the reciprocal fusion genes is shown for patients where balanced reciprocal fusion genes were detected. Expression levels were measured

Supplementary Figure 5

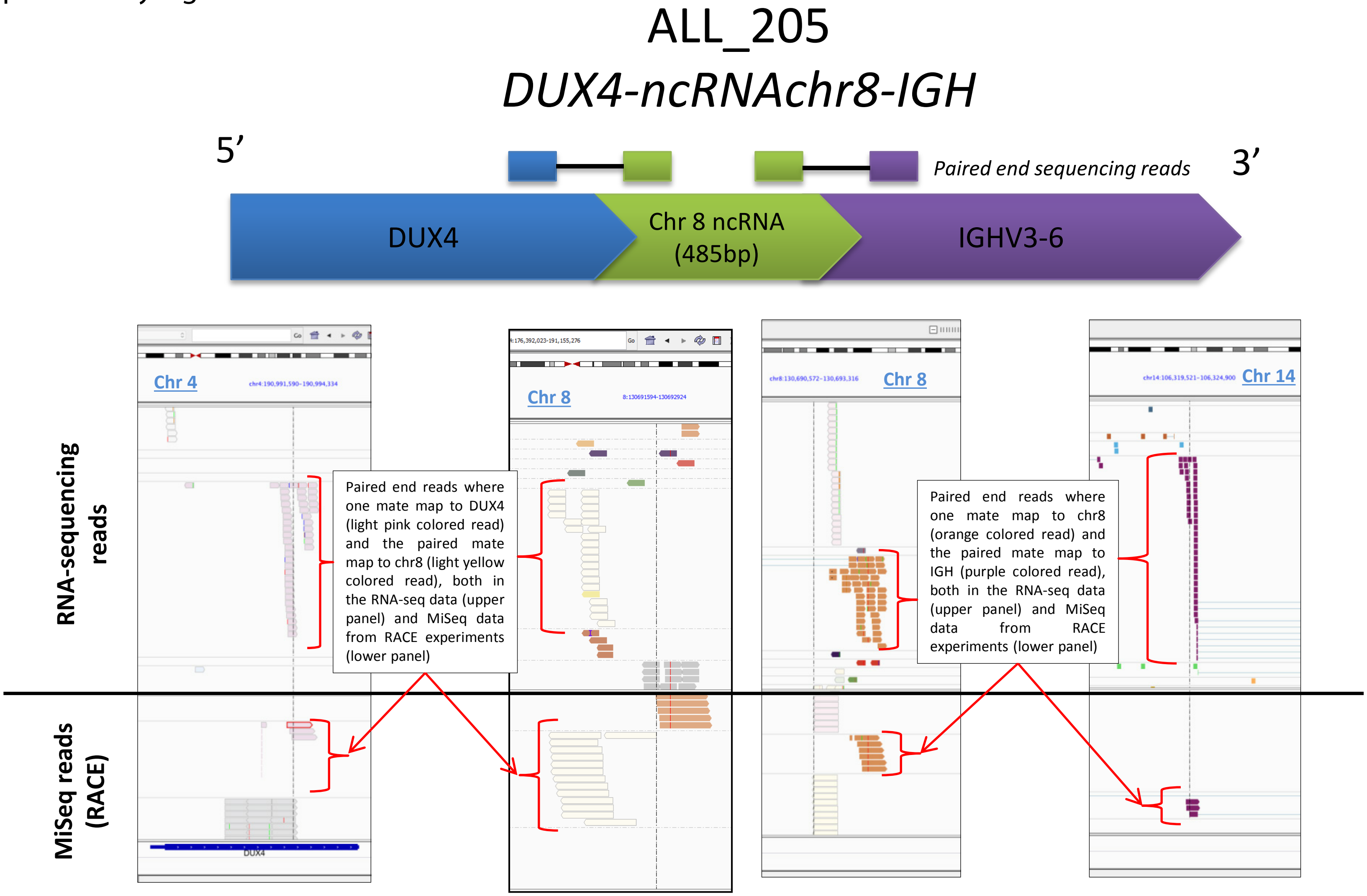

Supplementary Figure 5A: Validation of the suspected DUX4-r patient ALL_205 by RACE. A *DUX4-ncRNAchr8-IGH* fusion gene was detected in ALL_205 and a schematic illustration of the fusion gene is shown on the top of the figure. Paired-end RNA-sequencing and MiSeq reads (from RACE experiments) that span across the fusion junction are visualized in IGV and shown in the lower part of this figure. Paired sequencing reads where one mate map to DUX4 (chr4) (first panel from the left) and the other mate map to chr8 (second panel from the left) are indicated with a red arrow. Similarly, paired reads mapping to same region on chr8 (third panel from the left) and the other mate map to IGH (chr14) (last panel to the right) in the RNA-sequencing and MiSeq data are indicated by red arrows.

Supplementary Figure 5

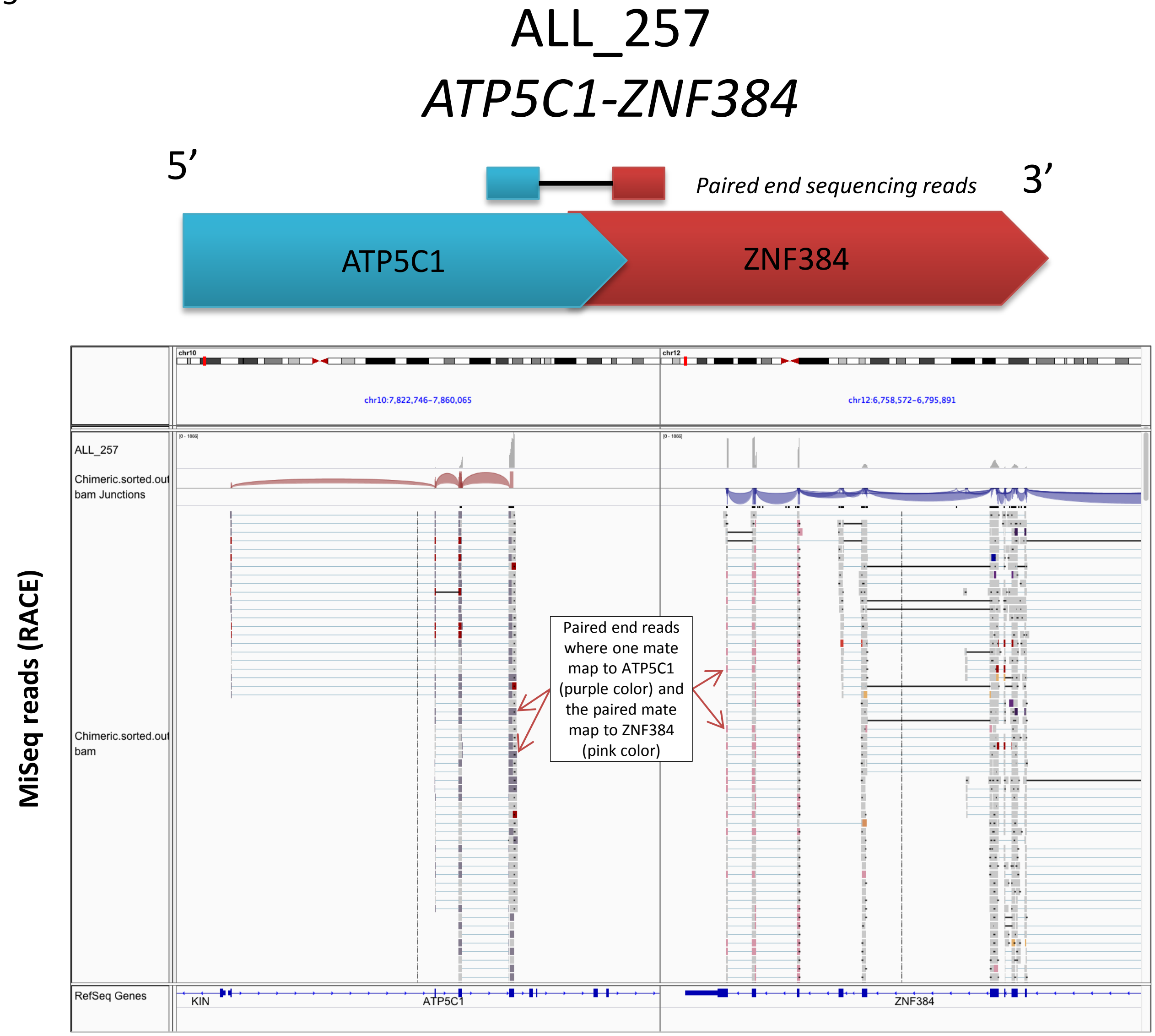

Supplementary Figure 5B: Validation of the suspected ZNF384-r patient ALL_257 by RACE. MiSeq data from RACE experiments revealed a *ATP5C1-ZNF384* fusion gene in ALL_257 and a schematic illustration of the fusion gene is shown on the top of the figure. Paired-end MiSeq reads from RACE experiments that span across the fusion junction are visualized in IGV and shown in the lower part of this figure. Several paired sequencing reads where one mate map to *ATP5C1* (chr10) (left panel – purple reads) and the other mate map to *ZNF384* (chr12) (right panel – pink reads)were detected.

Supplementary Figure 6

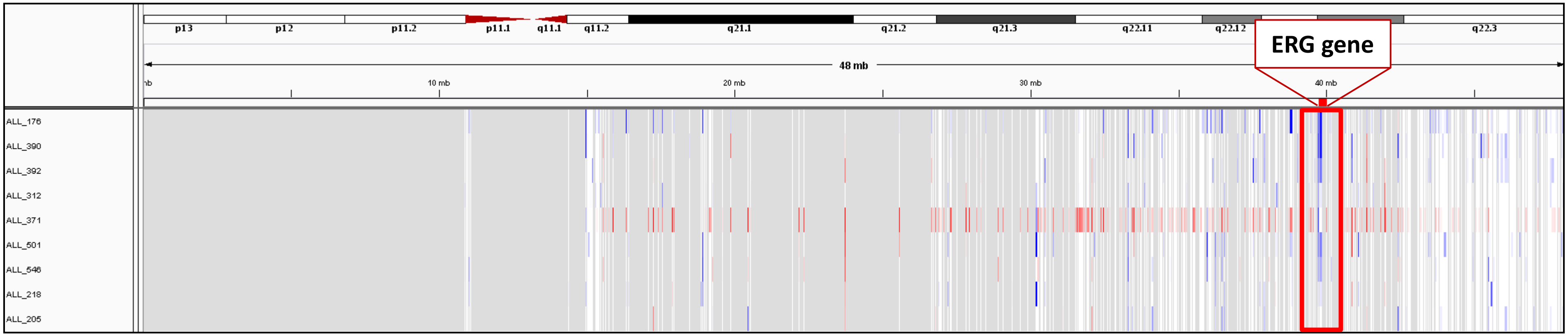

Supplementary Figure 6: ERG status for the nine *DUX4-IGH* patients. Copy number alterations (CNA) were detected using probe intensity levels from the Infinium HumanMethylation450 BeadChip. LogR ratios were calculated from probe intensity levels by normalization and transformation of intensities for sample versus control, and used to screen for CNA using the Integrative Genomics Viewer (IGV). The ERG gene is highlighted with a red horizontal bar. Probes that are deleted (negative LogR ratio; <0) are colored blue and probes that are amplified (positive LogR ratio; >0) are colored in red.

Supplementary Figure 7

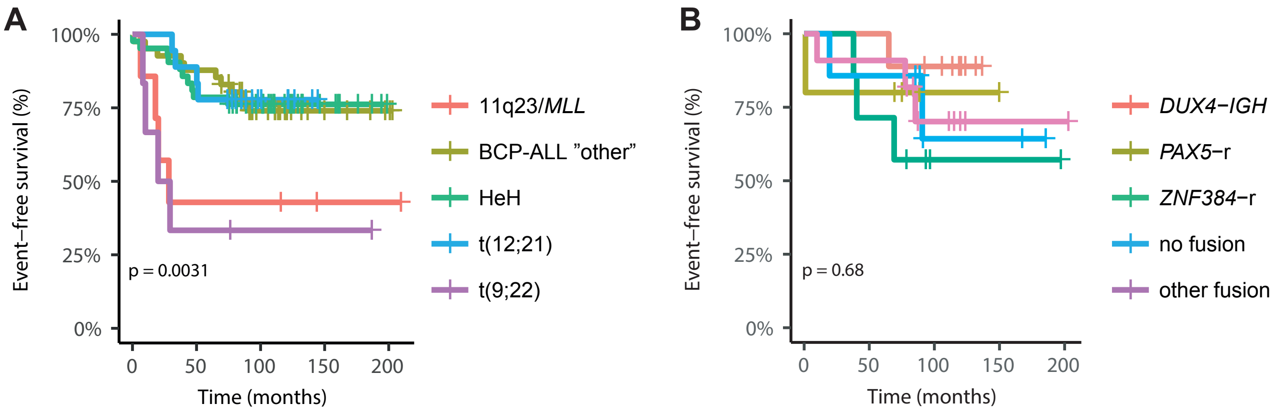

Supplementary Figure 7: Event-free survival in BCP-ALL patients with recurrent fusion genes. Kaplan-Meier estimates of the event-free survival of the recurrent cytogenetic subgroups of BCP-ALL (A) and in BCP-ALL "other" patients grouped by fusion genes (B). The group of patients denoted as BCP-ALL "other" at diagnosis had a favorable event-free survival rate similar to HeH and t(12;21) patients (A). However, when analyzed separately, different outcomes were observed in patients harboring the different fusion genes within the BCP-ALL "other" group (B). The time in months from diagnosis is plotted on the x-axis. The p-value was determined with the log-rank test.
